# Heat shock and prolonged heat stress attenuate neurotoxin and sporulation gene expression in group I *Clostridium botulinum* strain ATCC 3502

**DOI:** 10.1371/journal.pone.0176944

**Published:** 2017-05-02

**Authors:** Katja Selby, Gerald Mascher, Panu Somervuo, Miia Lindström, Hannu Korkeala

**Affiliations:** Department of Food Hygiene and Environmental Health, Faculty of Veterinary Medicine, University of Helsinki, Helsinki, Finland; Institut Pasteur, FRANCE

## Abstract

Foodborne pathogenic bacteria are exposed to a number of environmental stresses during food processing, storage, and preparation, and in the human body. In order to improve the safety of food, the understanding of molecular stress response mechanisms foodborne pathogens employ is essential. Many response mechanisms that are activated during heat shock may cross-protect bacteria against other environmental stresses. To better understand the molecular mechanisms *Clostridium botulinum*, the causative agent of botulism, utilizes during acute heat stress and during adaptation to stressfully high temperature, the *C*. *botulinum* Group I strain ATCC 3502 was grown in continuous culture at 39°C and exposed to heat shock at 45°C, followed by prolonged heat stress at 45°C to allow adaptation of the culture to the high temperature. Growth in continuous culture was performed to exclude secondary growth phase effects or other environmental impacts on bacterial gene transcription. Changes in global gene expression profiles were studied using DNA microarray hybridization. During acute heat stress, Class I and III heat shock genes as well as members of the SOS regulon were activated. The neurotoxin gene *botA* and genes encoding the neurotoxin-associated proteins were suppressed throughout the study. Prolonged heat stress led to suppression of the sporulation machinery whereas genes related to chemotaxis and motility were activated. Induced expression of a large proportion of prophage genes was detected, suggesting an important role of acquired genes in the stress resistance of *C*. *botulinum*. Finally, changes in the expression of a large number of genes related to carbohydrate and amino acid metabolism indicated remodeling of the cellular metabolism.

## Introduction

The Gram-positive, endospore-forming, obligate anaerobe bacterium *Clostridium botulinum* is the major causative agent of the severe, potentially lethal, neuro-paralytic disease botulism, which is induced by botulinum neurotoxin (BoNT) formed by the bacterium during vegetative growth [1,2]. BoNT inhibits release of the neurotransmitter acetylcholine at mammalian nerve endings, leading to flaccid paralysis. It is one of the most toxic substances known to mankind, with an estimated amount of less than 100 ng causing human death and possessing high potential for abuse as a bioterrorism agent [3]. Botulism can occur after oral, inhalational or iatrogenic intake of BoNT, or upon toxin formation in the human body after infection with *C*. *botulinum* spores (wound and intestinal form). The predominant forms are foodborne botulism, which is an oral intoxication with BoNT formed by *C*. *botulinum* in food or drink, and infant botulism, which is a form of intestinal botulism affecting children at an age of under one year probably due to their premature competitive intestinal microbiota [1]. In the recent years also injecting-drug-related wound botulism has become a more frequent form [4]. The highly resistant spores of *C*. *botulinum* are commonly found in the environment, especially in soil and marine sediments, and can therefore contaminate many food materials [5]. This ubiquitous nature of *C*. *botulinum*, the severity of the disease in humans and animals, and the high costs for treatment and outbreaks related to commercial food products make the bacterium a major concern for public health and the food industry [6,7].

Although studied for decades, the mechanisms of regulation and control of growth and neurotoxin production in *C*. *botulinum* remain poorly understood. Environmental as well as metabolic signals have been discussed to affect the regulation of toxin production [8–11]. During growth in food or in the tissue or intestine, *C*. *botulinum* is exposed to a number of environmental stresses like changes in temperature, pH and osmolarity, reactive oxygen species, and starvation for nutrients. Even though the bacterium encounters variable stresses in different environments, the effects of the stresses on the bacterial cell and its metabolism are expected to show some similarity as most stresses predominantly damage and impair the function of proteins and bacterial DNA [12]. For the Gram-positive model organism *Bacillus subtilis* it has been shown that heat shock, a sudden up-shift in environmental temperature, induces general stress response proteins as well as a smaller set of heat stress specific proteins [13,14]. Many of these general stress response proteins are conserved molecular chaperones and proteases that ensure a protein quality control system for the cell. Their induction can result into better tolerance of subsequent stresses of other nature, the so-called ‘cross-protection’, which is a special risk for the food industry when applying the hurdle technology to control foodborne pathogens [13–15]. Many genes coding for conserved general stress proteins are present in *C*. *botulinum* [16]. Class I heat shock genes were found to be up-regulated after heat shock in *C*. *botulinum* and insertional mutations of *hrcA*, their regulator gene, or *dnaK*, encoding a chaperone, led to deteriorated growth at high temperature and under pH or salt stress [17,18]. In addition to these mechanisms, a DNA microarray study on a mid-exponential *C*. *botulinum* batch culture revealed heat shock-induced transcriptional changes in genes related to the translational machinery and protein biosynthesis, transcriptional regulators, and flagella biosynthesis [17]. However, information on growth-phase-independent heat stress response and mechanisms of long-term adaptation of *C*. *botulinum* to stressful environments is not available.

Here we provide new insights into the global heat shock response and the cellular mechanisms of adaptation to prolonged heat stress in *C*. *botulinum* grown in continuous culture, and discuss their possible implications in *C*. *botulinum* physiology. Utilizing continuous culture instead of a batch culture allows to uncouple transcriptional responses induced by high temperature stress from growth-phase-dependent or secondary responses due to conditions like pH stress or starvation [19]. Continuous bacterial cultures have been successfully used in study of clostridial metabolism for example in the solvent-producing *Clostridium acetobutylicum* [20,21]. Understanding the mechanisms *C*. *botulinum* uses to cope with environmental stresses is important when developing novel effective measures to control the growth and toxin production by this bacterium in foods and in the human body, which represent stressful environments.

## Results and discussion

### Growth of *C*. *botulinum* strain ATCC 3502 in continuous culture

**Fig 1:**
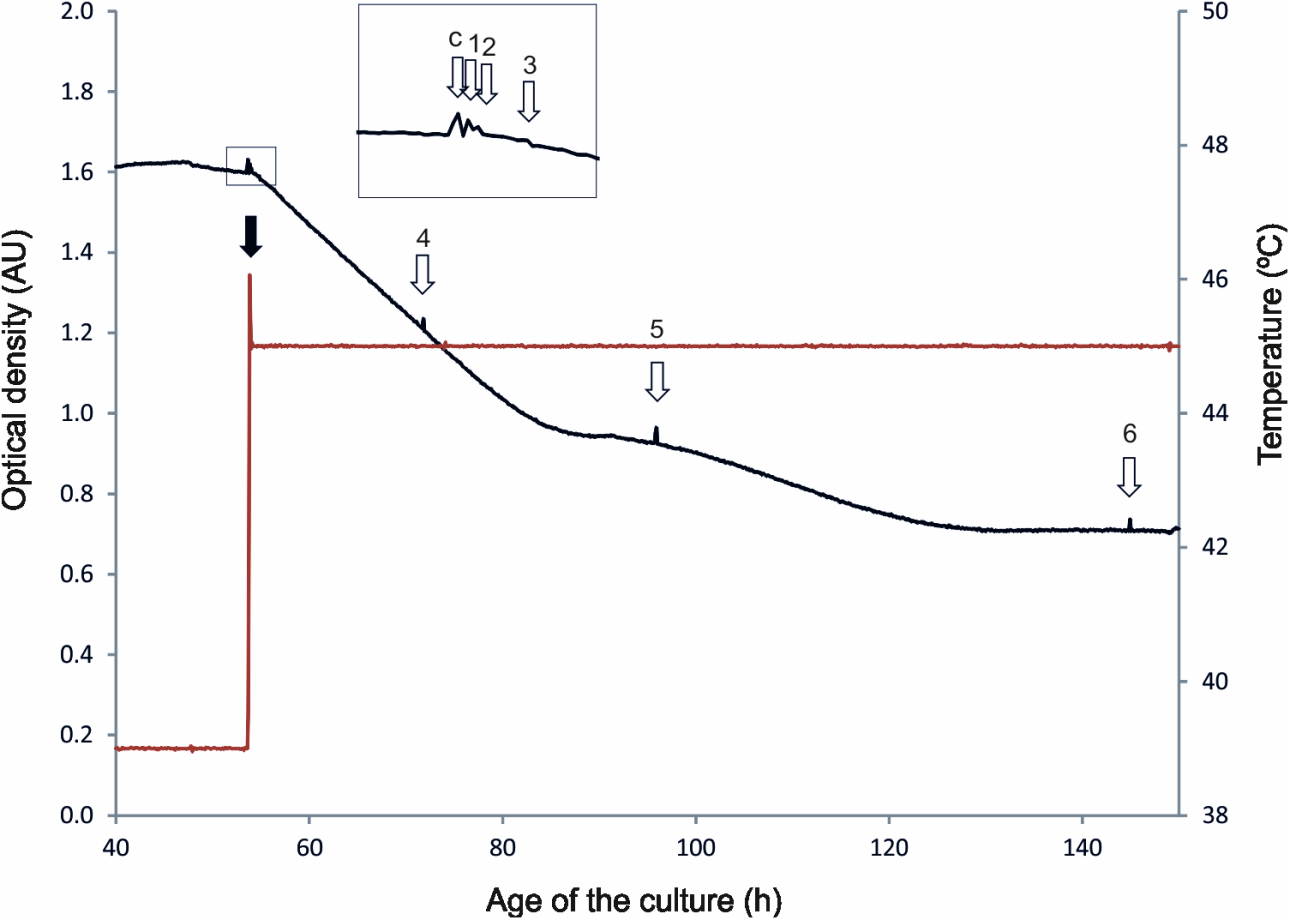
Growth curve of *Clostridium botulinum* ATCC 3502 exposed to heat shock at 45°C after growth at 39°C in continuous culture. Black line: optical density in absorption units (AU), measured at 600 nm; red line: temperature in°C. White arrows: sampling time points, c: control, 1: immediately after heat shock, 2: 10 min, 3: 1 h, 4: 18 h, 5: 42 h after heat shock, 6: adapted culture; black arrow: temperature up-shift.

*C*. *botulinum* ATCC 3502 was grown in continuous culture to study the impact of temperature stress on gene expression of the bacterial cells after heat shock and during adaptation to 45°C, compared to pre-heat-shock growth at 39°C. The advantage of continuous over batch culture of microorganisms for functional genomic studies is the possibility to analyze changes in transcription profiles specifically due to extrinsic stimuli, independent of changes due to intrinsic factors like growth phase, metabolic and acidic stress, or starvation [19]. The culture was brought to continuous growth at 39°C, with an OD_600_ stabilized to 1.6 to 1.7 AU. After temperature up-shift set to 45°C, the culture temperature increased shortly to 46°C during 4 min after the heat shock, and stabilized at 45°C by the 10-min time point. It took about 80 h of growth for the culture to adapt to the elevated temperature before stabilizing at a new OD_600_ of around 0.7 AU (Fig 1). Samples were withdrawn at 39°C before temperature up-shift, immediately when the culture temperature reached 45°C, 10 min and 1 h thereafter, during the adaptation to high temperature 18 and 42 h after heat shock, and after the culture adapted to 45°C and resumed continuous growth at stable OD (Fig 1).

### Identification of genes in *C*. *botulinum* affected by heat stress

**Fig 2:**
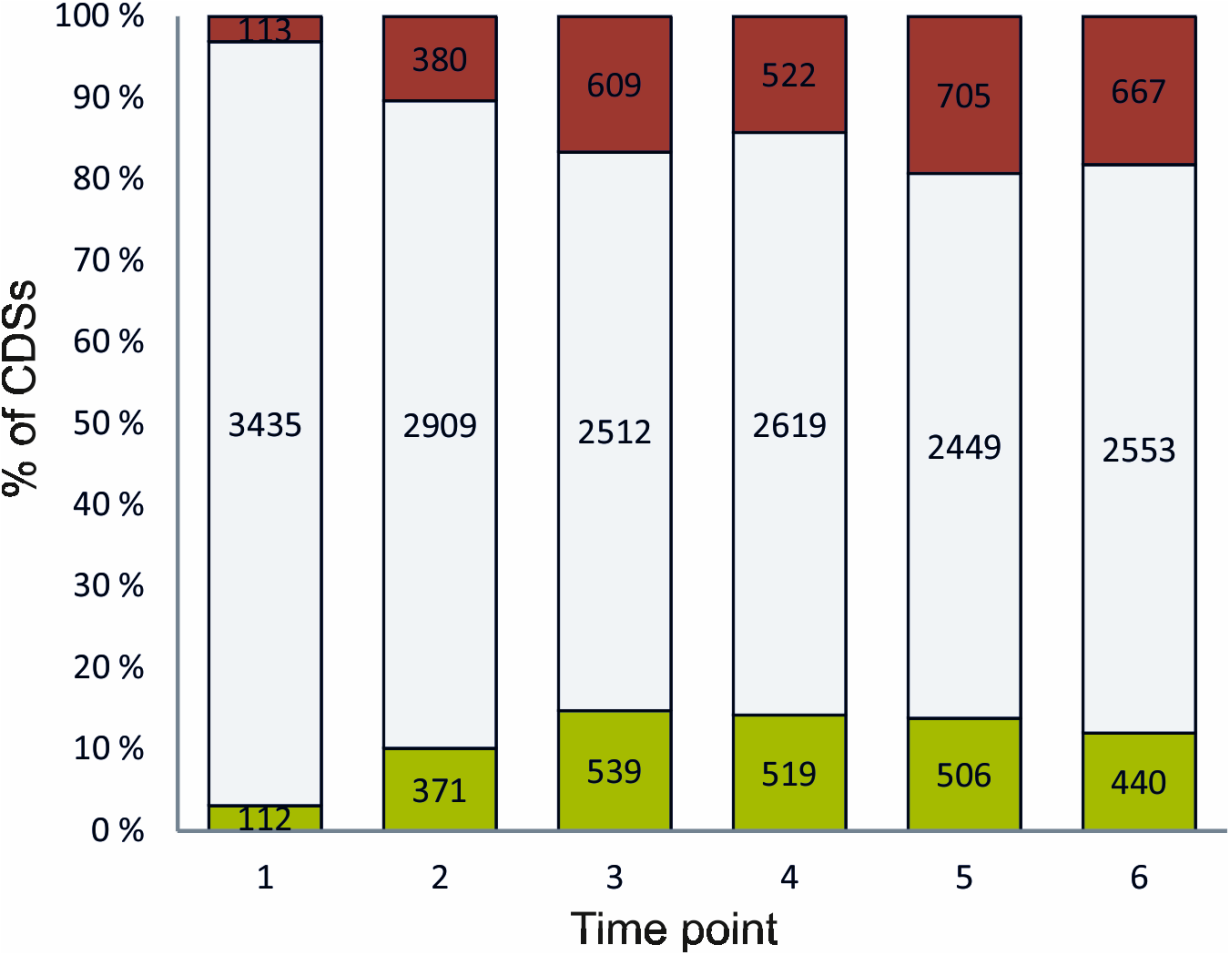
Number of CDSs of *Clostridium botulinum* ATCC 3502 being significantly up-regulated (red), down-regulated (green) or unaffected (grey) after exposure to high temperature at different time points after heat shock. 1: immediately after heat shock, 2: 10 min, 3: 1 h, 4: 18 h, 5: 42 h after heat shock, 6: adapted culture.

Transcriptomic analysis was performed to study the response of *C*. *botulinum* ATCC 3502 to heat stress caused by temperature up-shift (heat shock) from 39 to 45°C and subsequent adaptation and growth at 45°C in continuous culture using DNA microarrays. Protein coding sequences (CDSs) were considered to be significantly affected by high temperature if their transcription levels changed at least 2.0-fold at one or more of the six time points after the temperature up-shift compared to continuous growth at 39°C (log_2_-ratio ≥ 1.0 or ≤ -1.0, false discovery rate [FDR] ≤ 0.05). CDSs with changes in gene expression of 4.0-fold or more were considered being strongly affected (log_2_-ratio ≥ 2.0 or ≤ -2.0, FDR ≤ 0.05). By meeting these criteria, 2204 of the 3660 studied CDSs were identified to be significantly and 936 to be strongly affected by high temperature at one or more time points. Heat shock had a significant impact on the expression of 225 CDSs immediately after temperature up-shift, of which 20 were strongly affected, and on the expression of 751 CDSs after 10 min, of which 171 were strongly affected (Fig 2). During subsequent growth at and adaptation to 45°C, the number of affected CDSs increased to 1148 after 1 h of growth and remained similar, with 1041 and 1211 genes being differently expressed after 18 h and 42 h of growth, respectively, and 1107 CDSs showing significant changes in expression in the continuous culture adapted to heat (Fig 2). The number of genes being strongly affected was 311 at 1 h, 460 at 18 h, 497 at 42 h of growth and 463 in the adapted culture.

### Clustering

**Fig 3:**
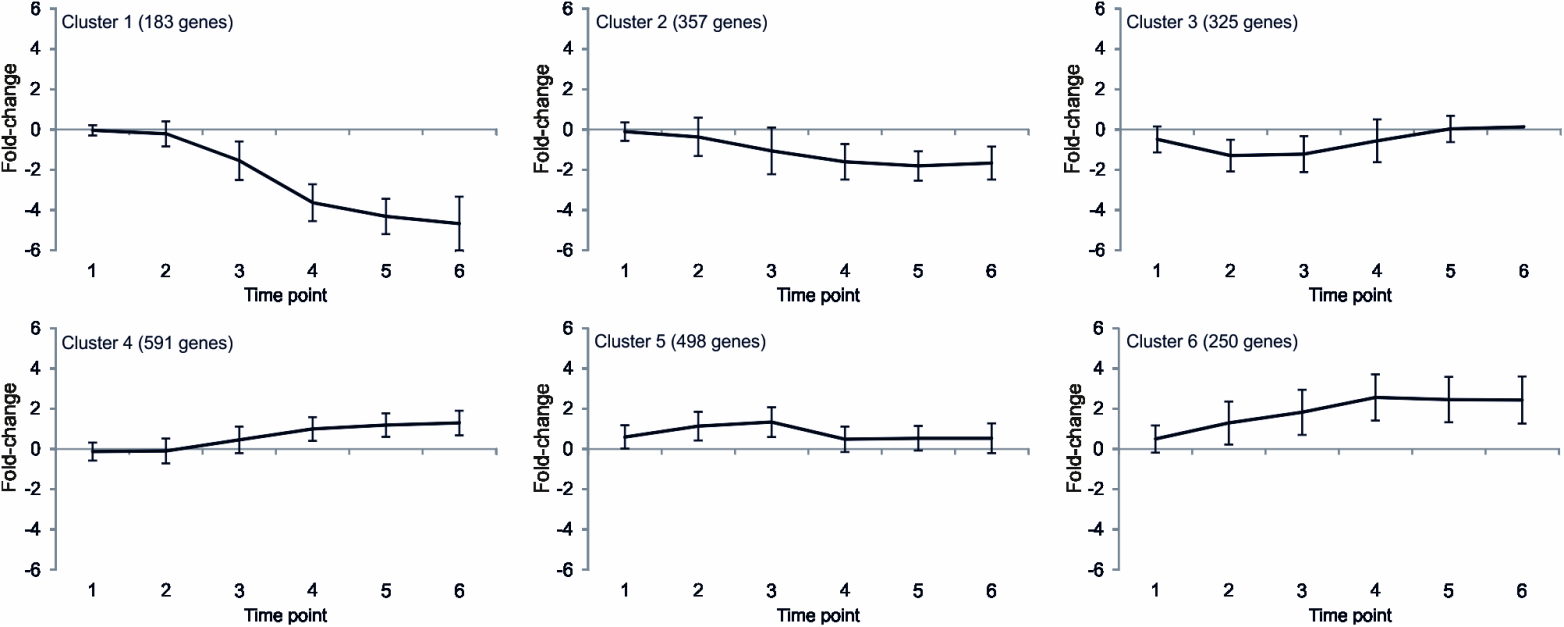
Gene expression profile of *Clostridium botulinum* ATCC 3502 showing the average fold-change in gene expression at 45°C compared to growth at 39°C of the significantly heat-affected genes assigned to clusters 1 through 6. In brackets: number of genes in cluster. Time point 1: immediately after heat shock, 2: 10 min, 3: 1 h, 4: 18 h, 5: 42 h after heat shock, 6: adapted culture. Error bars: standard deviation.

The CDSs of *C*. *botulinum* ATCC 3502 considered to be affected by high temperature grouped into six clusters using the k-means clustering method with Euclidean distance (Fig 3). Genes primarily affected during the early time points after temperature up-shift were considered to play roles in acute response, and fell in two clusters (cluster 3 and 5 consisting of 325 and 498 genes, respectively). Four other clusters consisted of genes being mainly affected during the later time points and thus considered to contribute to adaptation to elevated temperature (cluster 1, 2, 4, and 6; containing 183, 357, 591, and 250 genes, respectively). A number of 865 CDSs were considered to be predominantly suppressed (clusters 1, 2, and 3), suggesting roles during growth under favorable conditions, whereas 1339 genes were mainly activated at high temperature (clusters 4, 5, and 6), suggesting roles in stress and adaptation. The number of genes in different clusters for each functional group is shown in S1 Fig.

### Validation of DNA microarray results with quantitative real-time reverse-transcription PCR (RT-qPCR)

**Fig 4:**
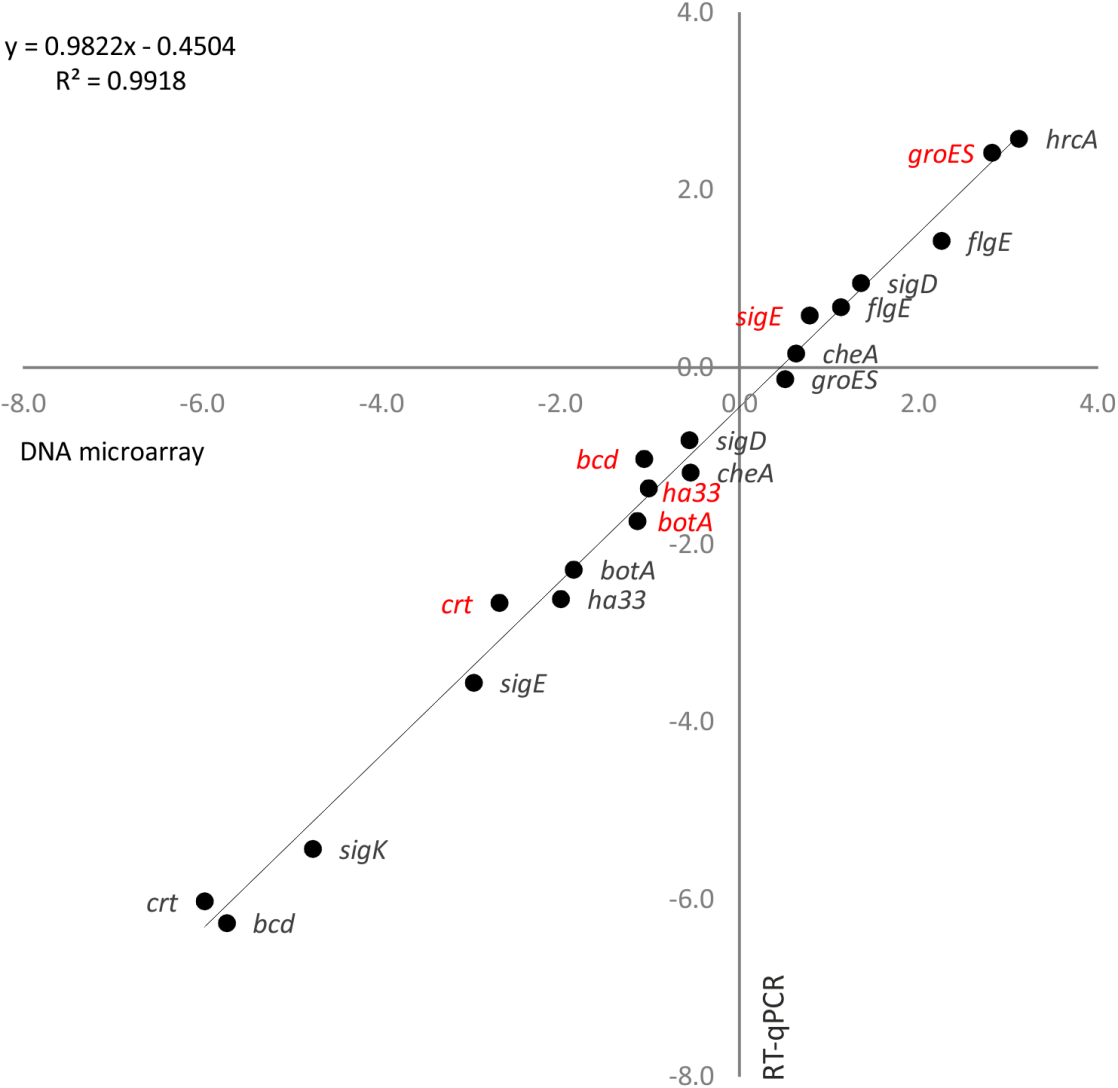
Validation of DNA microarray results using quantitative real-time reverse-transcription PCR (RT-qPCR). Log_2_ fold-changes of transcript levels measured with DNA microarrays (x-axis) and RT-qPCR (y-axis) in *C*. *botulinum* ATCC 3502 continuous culture 10 min (red) and 42 h (black) after temperature up-shift from 39 to 45°C. 16S *rrn* transcript levels were used as a normalization reference in the RT-qPCR. Linear regression analysis showed an R^2^ correlation value of 0.99 between the microarray and RT-qPCR transcription fold-change results.

Changes in gene expression after temperature up-shift determined by DNA microarray analysis were validated for a selection of genes at two different time points by relative gene expression analysis using RT-qPCR. The gene selection covered heat shock genes (*hrcA*, *groES*) and genes related to BoNT (*botA*, *ha33*), sporulation (*sigK*, *sigE*), motility (*sigD*, *cheA*, and *flgE*), and carbon metabolism (*cbo3199*, *cbo3202*), and included genes showing significantly activated or suppressed, or unaffected transcription levels in the microarray analysis. A linear regression analysis revealed a strong correlation between the log_2_ fold-changes detected with the two methods (R^2^ = 0.99) (Fig 4). The RT-qPCR method consistently gave slightly lower log_2_ fold-changes than the microarray method, which is likely explained by the different normalization approaches applied. Of the validated genes, only the expression of two genes at one time point each would be differently interpreted using the two methods: *cheA* would be determined as significantly down-regulated by RT-qPCR but only mildly down-regulated in the microarray analysis at 10 min after heat shock, and the expression of *flgE* as mildly instead of significantly up-regulated. However, since all the gene systems affected by high temperature and thus considered to be relevant for the current study involved multiple genes and time points, these minor inconsistencies between DNA microarray analysis and RT-qPCR data for single genes were considered negligible. Therefore we conclude that our DNA microarray analysis provides a reliable method for whole-genome transcriptional analysis. This is further confirmed by the fact that CDSs annotated as homologues of the *B*. *subtilis* heat shock stimulon genes [13] were up-regulated, whereas cold shock CDSs primarily activated at low temperature [22], were either suppressed or unaffected by heat.

### Heat-affected genes and their predicted functions in *C*. *botulinum*

The expression levels of selected genes significantly affected by high temperature are shown in S1 Table, and those of all genes are shown in S2 Table.

#### Transcription and translation

Exposure of an ATCC 3502 continuous culture to heat shock led to transient suppression of the prokaryotic translation machinery, indicating a temporal growth arrest of the culture. Amongst the suppressed genes were those coding for 30S or 50S ribosomal proteins, aminoacyl tRNA synthetases required for charging tRNA with their corresponding amino acids, as well as initiation and elongation factors. A majority of 41 of the 55 *C*. *botulinum* ATCC 3502 CDSs coding for 30S or 50S ribosomal subunits showed transient down-regulation 10 min and 1 h after temperature up-shift to 45°C (cluster 3), with 20 genes being strongly affected at 10 min. Also 14 of 24 genes encoding aminoacyl tRNA synthetases were affected and showed strong down-regulation 10 min and 1 h after heat shock (cluster 3). *infA* and *infC* encoding translation initiation factors and *efp*, *cbo2434*, *fusA*, *tufA*, and *tufB* encoding translation elongation factors were down-regulated 10 min after heat shock. Furthermore, genes encoding the DNA-directed RNA polymerase chains alpha (*rpoA*), beta (*rpoB*), and beta’ (*rpoC*) and *sigA*, coding for the”housekeeping sigma factor” A, showed significantly reduced expression 1 h after temperature up-shift (cluster 3), demonstrating a temporary block of transcription and translation after heat shock before restoring growth. This finding is in line with a study profiling gene expression of *C*. *botulinum* grown in batch culture, also suggesting growth arrest of the culture shortly after heat shock [17]. Such an arrest may allow the cells to prioritize mechanisms essential for survival at heat stress.

#### Heat shock proteins and chaperones

Temperature up-shift from 39 to 45°C induced CDSs annotated as homologues of the *B*. *subtilis* heat shock stimulon genes [13], which confirmed that our experimental set-up efficiently induced a heat shock response in *C*. *botulinum*.

Class I heat shock chaperons, together with ATP-dependent proteases, make a protein quality control system for bacterial cells. The HrcA-regulated *dnaK* and *groE* operons, consisting of class I heat shock genes (*hrcA*, *grpE*, *dnaK*, *dnaJ* and *groEL*, *groES*), code for molecular chaperons assisting in protein folding, a key to coping with heat denaturation of proteins. Both operons were up-regulated 1.7- to 5.6-fold immediately after temperature up-shift and remained strongly over-expressed until 1 h after heat shock (cluster 5). Highest transcription levels of class I heat shock genes were observed 1 h after heat shock, which, in line with our previous batch culture study [18], suggested a prolonged induction compared to *B*. *subtilis* [23,24]. The *groE* operon was more strongly induced than the *dnaK* operon, and, as opposed to the *dnaK* operon, its up-regulation continued at all later time points (1.4- to 2.5-fold). This finding emphasizes that the GroELS chaperone complex plays not only a role in the sudden heat shock response of *C*. *botulinum* but is also important for ensuring correct folding of proteins during adaptation to and growth at high, stressful temperature.

The *clpC* operon (*cbo3512-3508*) encoding homologues of the CtsR-regulated class III heat shock genes in *B*. *subtilis* was up-regulated 4.1- to 14-fold immediately after heat shock and reached massively increased expression levels of 17- to 45-fold at 10 min and 18- to 43-fold at 1 h after heat shock (cluster 6). Moreover, this operon remained expressed at higher levels (2.4- to 4.5-fold) during and after adaptation to 45°C than at 39°C. This contrasts to batch culture-grown *C*. *botulinum* undergoing heat shock where no induction of class III heat shock genes was detected [17]. Interestingly, *clpP*, which is essential for growth at high temperature for *B*. *subtilis* [25], did not show any significant heat induction in our study. This gene is thus likely to be regulated independently of CtsR and thereby probably not associated with heat stress response in *C*. *botulinum*. Moreover, while many other genes annotated as heat shock genes (e.g. *htpG*, *cbo1760*, and *clpB*) expectedly showed significant induction after temperature up-shift in our continuous culture, *cbo0831*, predicted to encode another heat shock protein, showed no activation and was repressed during heat adaptation and in the adapted culture. Thus this gene appears to lack a heat shock related function in *C*. *botulinum*.

The SOS response, a global cellular response to DNA damage under the control of the LexA transcriptional repressor (reviewed by Butala et al. [26]), is activated in *C*. *botulinum* subjected to cold shock [27]. Transcription of *recA*, encoding a protein that initiates the cleavage of LexA which leads to de-repression of the SOS system, was up-regulated from 10 min after heat shock onwards. In agreement, we found increased expression of several genes (*dinB*, *pcrA*, *ligA*, *uvrC*, and both copies of *dnaE*) homologous to those involved in the DNA repair processes of *B*. *subtilis* [28]. These findings show that *C*. *botulinum* utilizes the SOS system in response not only to cold but also to heat stress, as an additional mechanism to activation of the heat shock stimulon members described above. Interestingly, activation of the SOS response has been linked to toxin A production in *C*. *difficile* [29].

#### Botulinum neurotoxin

**Fig 5:**
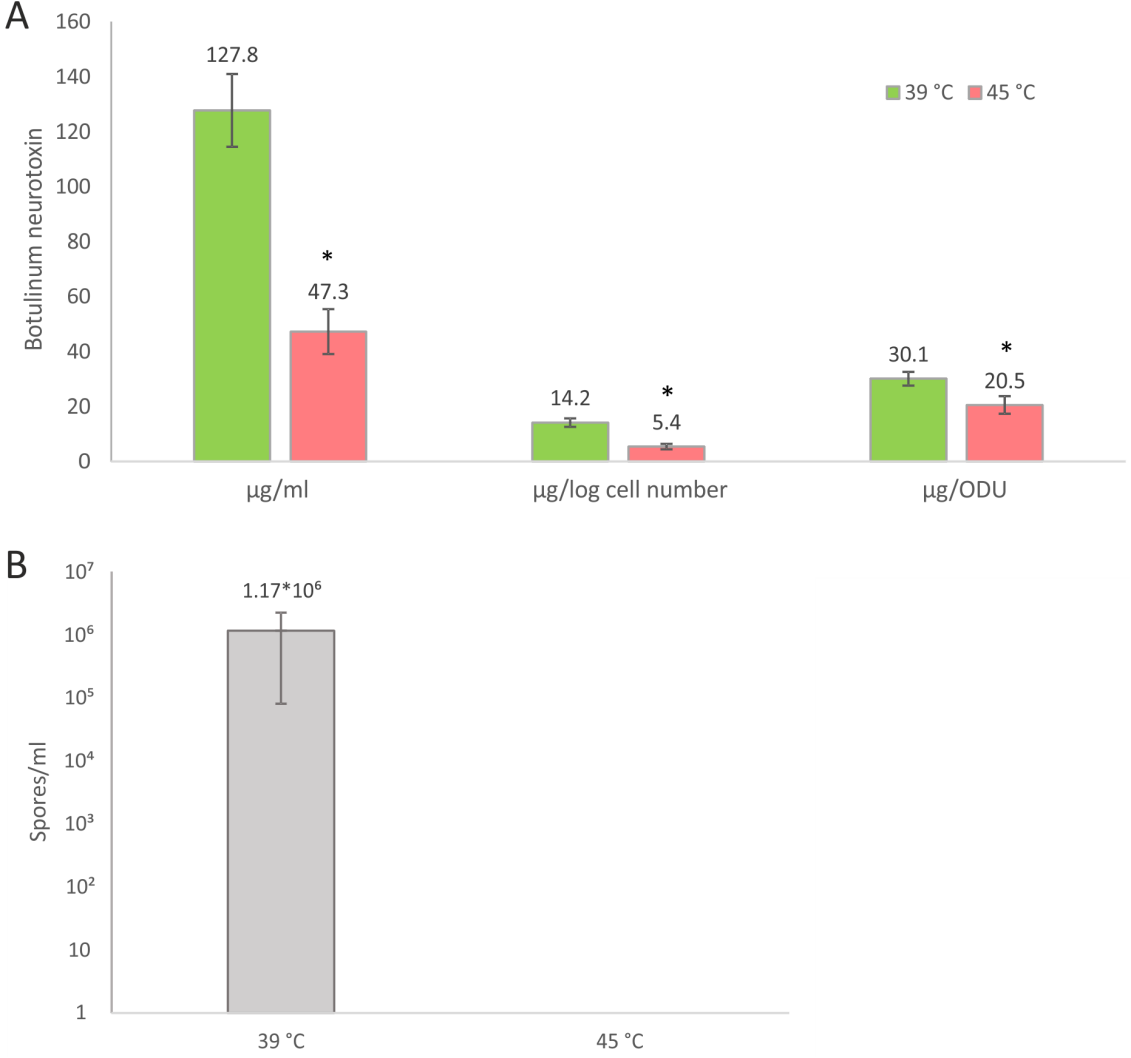
**Batch culture growth of *Clostridium botulinum* at 45**°**C resulted in reduced toxin production (A) and abolished sporulation (B) compared to growth at 39**°**C.** A. Concentrations of botulinum neurotoxin A (BoNT/A) detected with ELISA after 18 h of growth at 39°C (green bars) and 45°C (red bars); B. Spore count after 96 h of growth at 39 and 45°C. Error bars: standard deviation of five biological replicates, *: statistically significant (p < 0.05) difference between 45 and 39°C.

The botulinum neurotoxin genes are localized on the *C*. *botulinum* ATCC 3502 chromosome in two adjacent operons (*ntnh*-*botA* and *ha33-ha17-ha70*), flanking *botR* whose product, the alternative sigma factor BotR, drives the transcription of the two neurotoxin operons. Both operons showed significantly repressed transcription levels from 10 min onwards: 5.2- to 7.5-fold at 1 h after heat shock, 3.0- to 4.6-fold during heat adaptation, and 5.1- to 6.8-fold in the adapted continuous culture, in relation to pre-heat shock growth at 39°C (cluster 2). Temperature-controlled transcriptional suppression might lead to reduced neurotoxin production during prolonged growth under heat stress, which is in agreement with an early study on *C*. *botulinum* strain Hall A grown in a fermenter showing strongly reduced toxin production during growth at 45°C compared to 35°C [30]. High temperature also plays a role in the expression of some other clostridial toxins: suppressed expression of toxins A and B in *C*. *difficile* and phospholipase C in *C*. *perfringens* were reported at supraoptimal temperatures [31–33]. We tested the impact of high temperature on BoNT production for batch culture grown *C*. *botulinum* using botulinum neurotoxin type A ELISA. In accordance with the continuous culture gene expression profile, we detected significantly (p < 0.05) lower levels of BoNT after 18 h of growth at 45°C than at 39°C in a batch culture (Fig 5A).

In stressful environments, metabolic processes with high energetic cost for bacteria, such as production of large protein toxins and toxin complexes, are likely repressed to maintain those cellular functions that confer survival. However, there is controversial information available on the impact of high temperature on NTC gene expression and BoNT production in *C*. *botulinum*: The transcription of *botA*, *botR* and *ha17* was considered to be independent of high temperature as their expression levels measured by real-time reverse transcription PCR did not differ between *C*. *botulinum* Hall A batch cultures grown at 37 or 44°C, and the toxin concentrations measured in late-exponential cultures were similar at both temperatures [34]. In contrast, expression of the Ha33 component of the NTC was elevated after high-temperature challenge of a *C*. *botulinum* batch culture, suggesting that Ha33 acts as a neurotoxin stabilizing heat shock protein [35]. Finally, a DNA microarray study on a *C*. *botulinum* ATCC 3502 batch culture subjected to heat shock from 37 to 45°C for 15 min showed mild, although non-significant, induction of two toxin cluster genes (*ha17* and *ha33*) during the exponential growth phase [17]. However, the experiment lacked a non-heat-shocked control and thus it is not clear if such an apparent mild up-regulation of the NTC genes in a batch culture was due to true heat induction or due to a rapid onset of the NTC gene expression between the mid-exponential and transition phases of growth [34]. Such growth-phase-dependent effects on gene expression were most likely avoided in our experimental setup when exposing a steady-state continuous *C*. *botulinum* culture to heat shock.

Transcription of the NTC genes in *C*. *botulinum* ATCC 3502 is directed by the alternative sigma factor BotR [36]. Furthermore, positive control of *botA* expression by the global regulator CodY was recently proposed [11]. Also two-component systems (TCS) have been linked to control of BoNT expression, both positively [37] and negatively [10]. In the present study, low transcription levels of *botR* yielded low signal intensities that exceeded the detection threshold with sufficient FDR values only at three time points. Unexpectedly, however, at 42 h after temperature up-shift *botR* was significantly up-regulated unlike the structural neurotoxin genes. Since both *botR* and *codY* expression levels, plus the TCS regulators *cbo0607*, *cbo1041*, and *cbo1968*, proposed to positively regulate the NTC genes [37], were either unaffected or up-regulated throughout our experiment in parallel with down-regulation of the NTC structural genes, we anticipate the presence of a heat stress-induced negative regulatory mechanism that suppresses BoNT production in *C*. *botulinum* ATCC 3502 independently of the known positive regulators. The hybridization signal strength for the *cbo0787/cbo0786* TCS genes, coding for the only negative regulator of BoNT expression proposed to-date [10], was too low in our data to allow reliable estimation of transcriptional fold-change at heat exposure. Very low signal strength may reflect inactivity of this TCS in our conditions and also speaks for an as-yet unknown system repressing BoNT production at high temperature. Several so far uncharacterized TCS genes showed up-regulation at high-temperature stress. Should these TCSs be involved in a larger network that represses NTC gene expression at high temperature, remains to be studied. Finally, concurrent suppression of *cbo0352*-*cbo0353* and the NTC genes provides further support for a recent suggestion that the TCS CBO0353/CBO0352 positively controls BoNT synthesis [37].

#### Sporulation

Toxin production in many pathogenic clostridia is growth phase dependent, and a temporal and regulatory link between toxigenesis and spore formation has been suggested for *C*. *perfringens* and *C*. *difficile* (reviewed by Al-Hinai et al. [38]). Also *C*. *botulinum* batch cultures induce their NTC gene expression in transition from exponential to stationary growth phase, in parallel with the onset of sporulation [34,39].

The expression of sporulation genes was strongly affected by temperature in our study: We found significant down-regulation of 56 out of the 88 CDSs assigned to the functional category of differentiation, sporulation, and germination. These genes were related to all stages of sporulation and were suppressed mostly from 1 h after heat shock onwards (clusters 1 and 2, S1 Fig), suggesting that *C*. *botulinum* omits spore formation at stressfully high temperature. A batch culture of *C*. *botulinum* ATCC 3502 confirmed this assumption: No spores were detected in heating assays after 96 h of growth at 45°C, whereas growth at 39°C resulted in spore levels as high as 10^6^ spores/ml (Fig 5B).

Parallel down-regulation of sporulation and NTC gene expression may suggest common regulation of the two traits. Nevertheless, essential repression of the sporulation machinery appeared to occur slightly later in relation to temperature up-shift than repression of the NTC genes. This raises intriguing questions of the physiological and evolutionary significance of the two traits and their selective advantages in stressful conditions, and warrants further study of the factors that motivate and regulate these traits.

That spore formation, a key characteristic making *C*. *botulinum* a particularly challenging food pathogen, seemed abolished at supraoptimal temperature contradicts with the idea that sporulation is a key strategy bacteria employ to endure unfavourable environmental conditions. Bacterial spores are generally considered dormant and highly resistant to e.g. desiccation, UV-light, radiation, and heat [40]. Temperature dependence of bacterial sporulation has been known for long, with the optimum sporulation temperature assumed to be close to the optimum growth temperature. Sporulation has been described to take place between 15°C and 45°C for “most of the ordinary bacteria” [41]. A possible reason for repressed sporulation during heat stress could be protein and DNA damage. Enzymes and regulators of the sporulation cascade, or the single structural spore proteins, could be heat labile, making thus assembly of the spores fail at high temperature. Furthermore, spores containing substantially damaged DNA might not be able to serve the purpose of evolution in which preservation of intact genetic information during the bad times and successful germination and outgrowth in the good times are essential. The *C*. *botulinum* cells in our experiment seemed to respond to high cellular levels of temperature-damaged proteins and DNA, not only by activation of stress response mechanisms, such as proteins related to DNA repair and DNA metabolism, molecular chaperons, and proteases, but also by repressing the highly energy-consuming sporulation cascade and probably switching to alternative survival strategies. This idea is supported by the fact that a batch culture of *B*. *subtilis* exposed to production and secretion stress, which also leads to protein misfolding, suppressed sporulation gene expression from early on [42].

In *B*. *subtilis*, DisA, which scans the bacterial chromosome for DNA integrity, delays the initiation of sporulation if high level of DNA damage is sensed [43]. The CDS *cbo3507*, coding for a DisA homologue, appeared to be co-transcribed with the class III heat shock *clpC* operon in our data. This operon is controlled by CtsR, considered to be the master regulator of protein control in Gram-positives, which has been shown to bind to its target DNA in a temperature dependent manner [44]. The connection between CtsR and DisA could serve as a link between heat stress and the suppression of sporulation in *C*. *botulinum*.

Interestingly, *sigK*, encoding the late mother cell specific sporulation sigma factor SigK in *B*. *subtilis*, was significantly suppressed already from 1 h after heat shock onwards, whereas the other sporulation sigma factor genes *sigF*, *sigE*, and *sigG* [45] were down-regulated mainly during and after heat adaptation. These findings support the proposed role of SigK in the control of early sporulation or even pre-sporulation events in clostridia [46–48]. A batch culture experiment revealed reduced expression levels of *spo0A*, encoding the sporulation master regulator Spo0A, in a *sigK* mutant and suggested that SigK regulates *spo0A* [48]. However, the temporal gene expression patterns observed in the current continuous culture did not support similar sequence of regulation: *sigK* was steadily and significantly suppressed 1 h post heat shock onwards, while *spo0A* was repressed only immediately after hat shock and in the heat-adapted culture. Further studies are required to unravel the putative interplay between SigK and Spo0A and to reveal if this interplay is dependent on growth phase or environmental conditions. In light of current knowledge, it is plausible that both regulators have not only sporulation-related but also other functions in the cell physiology. Indeed, a functional SigK is required for optimal growth of *C*. *botulinum* at low temperature and in osmotic stress [49], and Spo0A has been attributed to regulation of virulence and biofilm formation in *C*. *difficile* [50,51].

Genes encoding the sporulation sigma factors (*sigE*, *sigF*, *sigG*, and *sigK*) were suppressed significantly in the heat-adapted culture. Their regulons in *C*. *botulinum* remain to be characterized but are likely to share similarities with corresponding regulons in other spore-formers. Indeed, a large proportion of genes homologous to members of the SigF, E, G, and K regulons in *C*. *difficile* [52] were suppressed under heat stress. As anticipated, these down-regulated genes included many annotated with sporulation-related functions, like stage II-V sporulation proteins, small acid soluble spore protein, spore-cortex-lytic protein, spore coat proteins, and spore maturation proteins. Genes that have not been related to sporulation in *C*. *botulinum* thus far included *cbo3395A* and *cbo3396*, homologous to genes encoding the putatively SigE-regulated toxin-antitoxin system MazF-MazE in *C*. *difficile* [52,53]. Another toxin-antitoxin system, SpoIISA/SpoIISB, plays a role in sporulation in *B*. *subtilis* [54]. Possible functions of *cbo3395A*/*cbo3396* or other toxin-antitoxin systems in the sporulation of *C*. *botulinum* warrant further studies. Additionally, four homologous genes (*cbo0900*, *cbo1594*, *cbo2267*, and *cbo2273*) with high similarity to the SigK-controlled *cd3620* encoding a protein with unknown function in *C*. *difficile*, were strongly down-regulated during heat stress response. The large number of highly similar gene loci with similar expression patterns in the *C*. *botulinum* ATCC 3502 genome suggests an important role for this gene and its product in *C*. *botulinum*. Finally, a small proportion of genes homologous to members of the sporulation sigma factor regulon in *C*. *difficile* were activated by high temperature. These genes are likely to have their primary roles in non-sporulation-related functions in *C*. *botulinum*, putatively contributing to heat stress response controlled by a different regulatory mechanism.

#### Chemotaxis and motility

More than half of the 84 genes of *C*. *botulinum* strain ATCC 3502 annotated to be chemotaxis- and flagella-related were activated by high temperature (clusters 4, 5, and 6; S1 Fig). Most of them are located in two loci on the ATCC 3502 chromosome. All genes of the first locus (*cbo2637*–*2666*) showed mostly significantly increased transcription at least at one time point during heat adaptation and in the culture adapted to 45°C. Furthermore, the 24 genes of the second chemotaxis- and flagella-related locus (*cbo2730*-*2753*) were mildly induced by high temperature, eight of them significantly. These results could suggest that *C*. *botulinum* utilizes motility as an adaptive response to prolonged high temperature stress, probably to escape harsh conditions and to search for a more beneficial environment. The assumed increase in motility at stressfully high temperature, however, could not be confirmed on low-agar swarming plates.

Increased motility by the use of flagella is a possible alternative response to unfavorable conditions in *B*. *subtilis* and in other spore formers [40]. To avoid entering the costly process of sporulation, motility could be exploited to occupy more beneficial environmental niches and to compete with other micro-organisms [40]. Motility and sporulation are both growth phase dependent traits in batch culture, and are assumed to be oppositely regulated [55–58]. Similarly, when a large proportion of sporulation related genes was suppressed in the continuous culture adapted to 45°C, we found significant activation of most chemotaxis and motility genes. These genes encoded chemotaxis proteins and different parts of the flagella, its assembly and biosynthesis machinery, and SigD, the flagellar operon specific sigma-28 factor. In *B*. *subtilis*, increased transcription of motility genes was described for cells under production and secretion stress [42]. Such a finding was explained by a relatively high level of SigD in stressed cells outcompeting scarce sporulation sigma factors, with subsequent up-regulation of motility and down-regulation of sporulation genes, as also observed in our experiment.

#### Prophages

The genome of *C*. *botulinum* ATCC 3502 harbors two unique large loci of genes assigned to bacteriophages and IS elements (*cbo1679–1755* and *cbo2394–2312*, further referred to as prophages 1 and 2, respectively) [16]. Their expression was affected by high temperature: Prophage 1 appeared to be strongly induced by high temperature primarily during the adaption phase and in the adapted culture (68 genes in clusters 4–6; S1 Fig). Prophage 2 appeared to be differently affected by high temperature: Most CDSs of its upstream half (*cbo2392*–*2361*) showed a significant increase in transcription from 1 h onwards and peaking during early adaptation (18 h) (cluster 6). The downstream half of prophage 2 (*cbo2360*–*2325*) was down-regulated throughout the study (1.4- to 14-fold reduction; clusters 2 or 3).

Transcriptional activation of many phage genes at high temperature could indicate an entry of lysogenic phages into the lytic cycle, leading to replication of phages and eventually to lysis of bacterial cells. Induction of the lytic cycle by heat has been described for prophages of *E*. *coli* and *B*. *subtilis* [59,60]. The observed reduction in our culture OD after temperature up-shift could indicate heat-induced cell lysis by activated prophages. However, since most prophage genes coding for structural phage proteins and proteins related to assembly of the phage tail or capsid were not activated by temperature up-shift, heat induction of the *C*. *botulinum* prophages and thus proliferation of intact phages seems unlikely.

Lysogenic phages integrated into the bacterial chromosome as prophages can change the bacterial phenotype, fitness, and pathogenicity by disruption of host genes at the insertion site or by transfer of beneficial genes into the host genome (reviewed by Bruessow et al. and Fortier and Sekulovic, [61,62]). In *B*. *anthracis*, the impact of prophages on biofilm formation, possibly increasing resistance to environmental stresses, and sporulation was reported and related to prophage encoded transcriptional regulators [63]. We found significantly increased expression of several prophage encoded regulators (S1 Table) from 10 min after heat shock onwards. The two strain-specific prophages could therefore have an impact on the phenotype of ATCC 3502, possibly contributing to its relatively high maximum growth temperature compared to other *C*. *botulinum* group I strains [64].

#### Carbon metabolism and fermentation

The genome of the proteolytic *C*. *botulinum* strain ATCC 3502 harbors all genes allowing the bacterium to utilize glucose through glycolysis and to ferment pyruvate, either through acidogenesis into acetate and butyrate, or through solventogenesis into butanol and ethanol [16]. Temperature up-shift led to strong suppression of fermentation pathway genes required for butanol and butyrate production (S1 Table), possibly leading to reduced levels of these compounds in heat stressed *C*. *botulinum* culture. Two of these genes (*cbo3202* and *cbo3199*) were linked also to low-temperature stress response of ATCC 3502 at 17°C [65]. Interestingly, butyrate in the growth medium has been shown to induce toxin synthesis by *C*. *difficile* [66]. Should butyrate have a similar impact on *C*. *botulinum* neurotoxin production, reduced expression of toxin genes in heat-stressed cells could be explained by low levels of butyrate produced in the culture.

Genes of the acetate production pathway were only mildly down-regulated soon after heat shock. In contrast, *cbo0345*, coding for an aldehyde-alcohol dehydrogenase, an enzyme that can convert acetyl-CoA to ethanol, was up-regulated 1 h after temperature up-shift onwards. It appears that *C*. *botulinum* grown in continuous culture might have shifted from butanol and butyrate to ethanol as fermentation end product soon after heat shock and retained this fermentation pattern during and after adaptation to high temperature. Interestingly, similar changes in metabolic pattern have been observed for a *C*. *difficile* strain with suppressed toxin production: Cysteine supplementation in the growth medium led not only to reduced toxin levels but also to strongly reduced levels of butanol and butyrate, whereas acetate levels remained unchanged and ethanol levels were elevated [66]. A strong link between clostridial fermentation metabolism and toxin production or possibly other traits seems evident and deserves further studies also in *C*. *botulinum*.

We found strong activation of several genes related to metabolism and transport of glycerol, trehalose and sorbitol soon after heat shock, with many of them remaining up-regulated during the adaptation and in the heat adapted culture (S1 Table). Glycerol is known as a compatible solute maintaining osmotic pressure in yeast cells and contributing to thermal stability of proteins through co-chaperone activity [67,68]. The other compatible solutes, trehalose and sorbitol, have been related to increased bacterial heat tolerance [69,70]. These sugars thus might play a role in the heat stress response of *C*. *botulinum* and its adaptation to heat, probably by enhancing protein stability.

#### Protein and amino acid metabolism

The genome of *C*. *botulinum* ATCC 3502 harbors several genes encoding secreted proteases contributing to the proteolytic nature of this strain [16]. The majority of these genes showed reduced transcription after temperature up-shift (S1 Table) indicating a drop of extracellular protein degradation activity and suggesting changes in nitrogen metabolism in response to high temperature stress.

The expression of some predicted ATCC 3502 amino acid fermentation pathways [16] changed during heat stress (S1 Table). Most genes coding for the glycine reductase complex (*cbo1257–1264*) were transiently suppressed after heat shock. Genes for the proline reductase complex (*prd* genes), in turn, were mainly activated up to more than 7-fold by prolonged exposure to high temperature (clusters 5 or 6), especially during and after heat adaptation. The glycine and proline reductase complexes enable *C*. *botulinum* to metabolize these amino acids in the so called Stickland reaction, a coupled oxidation–reduction reaction performed by many clostridia, characterized by reduction of one amino acid (electron acceptor) coupled to the oxidation of another amino acid (electron donor) [16,71]. The genes *cbo3292* to *cbo3289*, related to phenylalanine fermentation, were up-regulated during the adaptation and in the heat-adapted *C*. *botulinum* continuous culture. Interestingly, phenylalanine could serve as another electron donor in the Stickland reaction, potentially coupled to reduction of proline [71]. Changes in the fermentation of these amino acids might therefore be utilized as an adaptation mechanism to high temperature stress in *C*. *botulinum*. Remodeling of the Stickland reaction pathways was also observed in cold-shocked ATCC 3502 culture and was suggested to contribute to redox balance control of the cells [27].

Several genes related to biosynthesis of the sulfur-containing amino acid methionine were induced by heat (S1 Table). Induction of these genes seemed to aim at increasing intracellular methionine levels to overcome a limitation in its endogenous supply. Also in *B*. *subtilis* heat shock led to increased transcription of genes implicated in methionine biosynthesis [72]. Methionine dependence of growth at high temperature was further shown in *E*. *coli* [73] and was related to temperature sensitivity of homoserine *trans*-succinylase encoded by the heat-inducible *metA* [73–75]. Furthermore, activation of the methionine pathway has been linked to acetate stress in *C*. *acetobutylicum* and *E*. *coli* and to superoxide stress in *B*. *subtilis* [76–78]. Therefore overcoming auxotrophy for methionine appears to be a critical factor in bacterial stress response.

In summary, heat-shocked *C*. *botulinum* ATCC 3502 employed members of the well-studied *B*. *subtilis* heat shock stimulon as well as those of the SOS response to resist high temperature stress in a continuous culture. Further, the culture underwent a temporal growth arrest, and appeared to adapt to heat stress by remodeling of many cellular functions that represented more than 30% of all genes. The neurotoxin related genes were soon repressed by high temperature, and the sporulation machinery seemed to be repressed during heat adaptation and in the adapted culture. In contrast, chemotaxis and motility were activated upon adaptation to high temperature. Increased motility might thus serve as a long-term response to high-temperature stress in *C*. *botulinum*, perhaps as an escape mechanism alternative for sporulation. High-temperature stress further affected the metabolism of the organism, with altered pyruvate and amino acid fermentation pathways serving as possible long-term adaptation strategies at heat stress.

## Materials and methods

### Strain and growth conditions

*C*. *botulinum* strain ATCC 3502 was grown in continuous culture in a 5-l Braun Biostat B fermenter (B. Braun, Melsungen, Germany) with a working volume of 2 l at 39°C, 200 rpm, in anaerobic tryptone-peptone-glucose-yeast-extract (TPGY) broth (50 g/l tryptone, 5 g /l peptone, 20 g/l yeast extract [Difco, BD Diagnostic Systems, Sparks, MD, USA], 4 g/l glucose [VWR International, Leuven, Belgium], 1 g/l sodium thioglycollate [Merck, Darmstadt, Germany]), buffered with 6.25 g/l NaH_2_PO_4_ and 5.45 g/l KH_2_PO_4_ (VWR International). The culture was initially inoculated using 10 ml of *C*. *botulinum* ATCC 3502 culture grown anaerobically in TPGY for 24 h from a spore stock. The optical density (OD) of the culture was automatically continuously measured at a wavelength of 600 nm and recorded in absorption units (AU). Feeding was started after an OD of 1.5 AU was reached. The dilution rate was set at 0.035 h^−1^. The culture was constantly stirred and flushed with N_2_ to assure anaerobicity. TPGY for feeding was freshly autoclaved and kept in airtight containers with N_2_ overlay. Resazurin sodium salt (1 mg/l; Sigma-Aldrich, Steinheim, Germany) was used as anaerobicity indicator. The pH was kept constant at pH 6.8 by automatic addition of 3 M KOH (Sigma-Aldrich). The foam suppresser Antifoam A (Sigma-Aldrich) was added in a concentration of 20 mg/l to the medium. The experiment was performed in duplicate.

### Heat shock and sampling

After the culture reached continuous growth at a stable OD at 39°C (OD of 1.6 to 1.7 AU, from about 24 h after feeding start onwards) a 5-ml control sample of the culture was taken and the temperature set to 45°C. After 8 min, the culture temperature reached 45°C and another 5-ml sample was withdrawn (immediately after heat shock), followed by further sampling after 10 min and 1 h, during the adaptation of the culture to 45°C (18 h and 42 h after heat shock), and after the culture stabilized with continuous growth at 45°C at a stable OD of 0.7 to 0.8 AU. All samples were immediately mixed with a chilled stop solution (900 μl of 99.6% ethanol and 100 μl phenol/ml [Sigma-Aldrich], 2 ml per 5 ml sample volume), and incubated on ice for 30 min, followed by centrifugation for 5 min with 5000 g at 4°C. The supernatant was removed and the cell pellets were immediately stored at -70°C until RNA purification. Each sample was withdrawn in duplicate to consider technical replication.

### RNA isolation

After lysis of the cell pellet in 1 ml of lysis buffer (25 mg/ml lysozyme and 250 IU/ml mutanolysin [Sigma-Aldrich] in Tris-EDTA buffer [pH 8.0, Fluka, Biochemika, Buchs, Switzerland]) for 30 min at 37°C, total RNA was extracted using a commercial spin column system (RNeasy Midi Kit, Qiagen, Hilden, Germany) with on-column DNase treatment (RNase-Free DNase Set, Qiagen) and a second DNase treatment using the Ambion DNA-free kit (Applied Biosystems, Life Technologies Corporation, Carlsbad, CA) according to the manufacturer’s instructions. RNA concentration and quality was determined optically by measurement of the absorption units at the wavelength of 260 nm (A_260_) using the NanoDrop 1000 Spectrophotometer (Thermo Fisher Scientific, Waltham, MA, USA) and by electrophoresis with the 2100 Bioanalyzer (Agilent Technologies, Santa Clara, CA) using Prokaryote Total RNA Nano chips.

### Cy3- and Cy5-labelled cDNA synthesis

An amount of 2 μg of total RNA from each sample was reverse-transcribed and labeled directly with fluorescent dye. The 30-μl RT reaction mixture contained 5 μg of random primers, 40 U RNaseOUT™ Recombinant Ribonuclease Inhibitor, 6 μl 5x first-strand buffer, 3 μl of 100 mM DTT, 400 U SuperScript™ III Reverse Transcriptase (Invitrogen, Life Technologies Ltd, Paisley, UK), 0.6 μl dNTP mix (25 mM dATP, 25 mM dGTP, 25 mM dTTP, 10 mM dCTP [Promega Corporation, Madison, WI, USA]), and 2 nmol Cy3-dCTP or Cy5-dCTP (GE Healthcare, Buckinghamshire, UK). Each mixture was incubated for 3 h at 46°C and stopped with 1.5 μl of 20 mM EDTA. After addition of 15 μl of 0.1 M NaOH the RNA was hydrolyzed for 15 min at 70°C, followed by neutralization with 15 μl of 0.1 M HCl. The labeled cDNA was purified using a DNA purification column (QIAquick PCR purification kit; Qiagen) and eluted into 44 μl of elution buffer (Qiagen).

### Transcriptomic analysis with DNA microarrays

Custom designed, *in situ*-synthesized DNA microarrays (8x15K; Agilent Technologies), successfully employed in *C*. *botulinum* gene expression analysis studies [27,65], were used. They covered 3,641 chromosomal (out of the total of 3,648) and all the 19 plasmid-borne CDS of the ATCC 3502 genome [16]. A number of 3 to 14 60-mer oligonucleotide probes were designed for each CDS, depending on CDS length.

An amount of 300 ng of Cy3-labeled and 300 ng of Cy5-labeld cDNA samples were mixed into a volume of 18.4 μl, and 2.3 μg of salmon sperm DNA (Invitrogen) was added. After denaturation for 2 min at 95°C, the samples were placed on ice, and 5 μl 10x blocking agent and 26 μl 2x RPMI hybridization buffer (both Agilent technologies) were added to the cDNA mixture before loading it onto the slide. The arrays were hybridized under rotation for 16 h at 65°C. After hybridization, the slides were washed according to the manufacturer’s instructions and dried. The cDNA of each sample of both biological replicates obtained from the culture growing at 45°C was hybridized against the control sample (reference design), using a dye swap for the technical replicates resulting in four arrays for each experimental time point. Furthermore, a dye swap for the control sample was included on every microarray slide to control dye bias.

The slides were scanned (Axon GenePix Autoloader 4200 AL scanner, Westburg, Leusden, The Netherlands) using a 5-μm resolution at a wavelength of 532 nm and 635 nm. After image processing with the Gene Pix Pro 6.0 software (Axon Instruments), the data were analyzed using the R limma package [79]. The foreground and local background intensities of each spot were identified by the mean and median pixel values of the spot, respectively. The “normexp” method with an offset value of 50 was used to subtract local background from the foreground signal [80]. The signal intensities measured in the Cy5 and Cy3 channels were converted into a logarithmic (log_2_) scale and normalized using the loess method [81].

### Validation of DNA microarray results with quantitative real-time reverse-transcription PCR (RT-qPCR)

#### cDNA synthesis

The Maxima First Strand cDNA Synthesis Kit for RT-qPCR (Thermo Fisher Scientific) was used to reverse-transcribe 500 ng of total RNA from each pre-heat shock, 10 min, and 42 h after heat shock sample into complementary DNA (cDNA) according to the manufacturer’s instructions. No-RT controls of six pooled RNA samples were incubated with the enzyme mix replaced by RNase-free water. The cDNA samples were serially diluted in RNase-free water and stored at -20˚C.

#### Quantitative real-time PCR (qPCR)

qPCR reactions were performed in duplicate for each cDNA sample using the Maxima SYBR Green qPCR chemistry (Thermo Fisher Scientific) according to the manufacturer’s instructions with primers for 11 *C*. *botulinum* genes to be tested and the reference gene (S3 Table) in a Rotor Gene Q Real Time Thermal Cycler (Qiagen). Each 25-μl reaction included 4 μl of diluted cDNA as template (1:100 for genes to be tested, 1:10000 for 16S *rrn*), primers in a final concentration of 0.3 μM, 12.5 μl 2x Maxima SYBR Green qPCR master mix, and water. For each master mix, one non-template control with cDNA replaced by water was included in the qPCR run to control master mix contamination. The following cycling protocol was applied: polymerase activation at 95°C for 1 min, 40 cycles with 95°C for 10 sec and 60°C for 20 sec with data collection at the end of each cycle, and a final extension step for 1 min at 60°C, followed by a melt curve analysis (from 60°C to 98°C, 0.5°C steps, 10 s). The primers (S3 Table) were designed using the Primer3-web 0.4.0 web-interface (http://primer3.sourceforge.net/webif.php) based on the sequence of the published *C*. *botulinum* ATCC 3502 genome [16] and obtained from Metabion International AG, Planegg, Germany. For each primer pair, standard curves were constructed from 10-fold serially diluted pooled cDNA to calculate the reaction efficiency and the quantification threshold for detection of fluorescence above background using the Rotor Gene Q series software version 2.3.1. All no-RT controls were subjected to qPCR with melt curve analysis using primers for 16S *rrn* and showed no evidence of DNA contamination of the RNA samples. Expression values of the tested genes in samples taken 10 min and 42 h after temperature up-shift were calculated relative to the levels in the continuous culture grown at 39°C using the Pfaffl method [82]. The 16S *rrn* was used as the normalization reference [34,39].

### Phenotypic tests

*C*. *botulinum* ATCC 3502 was routinely grown in TPGY broth in anaerobic jars under anaerobic atmosphere created by AnaeroGen sachets (Oxoid Limited, Hampshire, United Kingdom). The strain was inoculated from spore stocks into 10 ml of TPGY broth and grown for 24 h at 39°C. This culture was re-inoculated into TPGY tubes, which were then incubated at 39 or 45°C to detect possible phenotypic differences between the two temperatures in toxin production and sporulation. The tests were performed with five biological replicates at each condition.

#### Toxin expression ELISA

In brief, to confirm the suppressive effect of elevated temperature on neurotoxin gene expression at the protein level, *C*. *botulinum* ATCC 3502 was grown at 39°C or 45°C and one ml of each culture was withdrawn after 18 h of growth. Samples were centrifuged at 8200 rpm for 10 min. The supernatants were directly used for ELISA analysis and the cell pellets were lysed prior to ELISA analysis by adding 1 ml of lysis buffer (10 mg/ml lysozyme [Sigma Aldrich] in 25 mM Tris-HCl [pH 7.5]) and incubating at 37°C for 1 h. BoNT/A concentrations were measured with commercially available botulinum neurotoxin type A ELISA kits (Tetracore, Rockville, MD) according to the manufacturer’s instructions. Purified BoNT/A complex (kindly provided by Michel R. Popoff, Institute Pasteur, Paris, France) was used to construct standard curves for quantification of BoNT in the samples.

#### Sporulation and viable cell count

To prove the suppressive effect of high temperature on sporulation gene expression at the phenotypic level, *C*. *botulinum* ATCC 3502 was incubated in TPGY broth for 96 h at 39°C or 45°C. At 96 h, one ml of each culture was sampled. Sporulation was studied after eliminating vegetative cells by heating the culture samples for 15 min at 80°C and observing growth after serial dilution of the heat-treated culture samples (1:10) in fresh TPGY broth. The spore concentrations (spores/ml) were calculated according to the Bacteriological Analytical Manual of the FDA (http://www.fda.gov/Food/FoodScienceResearch/LaboratoryMethods/ucm109656.htm). Total viable cell counts were determined similarly, excluding the heat treatment.

### Statistical analysis

Statistical analysis was performed to discover genes differently expressed in *C*. *botulinum* between 39 and 45°C. The R limma package was used [79]. Each of the multiple probes representing one CDS was analyzed separately using a moderated t-test with empirical Bayes variance shrinkage (“eBayes” function). Using a Benjamini-Hochberg adjustment (“topTable” function), the obtained P values were translated into FDR values [79]. FDR values are adjusted p-values allowing fewer false positive results in microarray expression analysis. The probe with a median unmodified p-value for the expression difference was selected to represent the CDS. Only CDSs with log_2_-ratio ≥ 1 or ≤ -1 and an FDR ≤ 0.05 were considered to be differentially expressed at 39 and 45°C and therefore to be affected by high temperature. These CDSs were included in further analysis.

The CDSs of *C*. *botulinum* ATCC 3502 considered to be affected by high temperature were grouped according to their time-dependent expression pattern during the exposure of the culture to high temperature. The open source software MultiExperimentViewer of the TM4 Microarray Software Suite was employed to create gene clusters using the k-means clustering method with Euclidean distance [83]. A number of six clusters was chosen to group the genes into biologically meaningful clusters.

The data discussed in this publication have been deposited in NCBI's Gene Expression Omnibus [84] and are accessible through Gene Expression Omnibus (GEO) Series accession number GSE73093 (http://www.ncbi.nlm.nih.gov/geo/query/acc.cgi?acc=GSE73093).

## Supporting information

S1 Fig Assignment of genes of *Clostridium botulinum* ATCC 3502 belonging to different functional classes into cluster 1 through 6 based on their expression profile after heat shock. The clusters were created using the k-means clustering method with Euclidean distance and are shown in Fig 3. Numbers indicate the number of genes assigned to each cluster. Green striped: cluster 1, green dotted: cluster 2, green solid: cluster 3, grey: no cluster, red striped: cluster 4, red solid: cluster 5, red dotted: cluster 6.
(TIF)

S1 Table Fold-changes in gene expression of selected genes with relevant biological functions of *Clostridium botulinum* strain ATCC 3502 at different time points after temperature up-shift to 45°C compared to continuous growth at 39°C and the assignment of these genes to a cluster based on their expression profile. Time points 1: immediately after heat shock, 2: 10 min, 3: 1 h, 4: 18 h, 5: 42 h after heat shock, and 6: adapted culture; values in bold: statistically significant change in expression (fold-change ≥ 2 or ≤ -2 and false discovery rate values [FDR] ≤ 0.05), values in italics: FDR values > 0.05; NA: no data available;–: no assignment to a cluster.
(DOCX)

S2 Table Log_2_ fold-changes in gene expression of all CDSs of *C*. *botulinum* ATCC 3502 under heat stress. Time points 1: immediately after heat shock, 2: 10 min, 3: 1 h, 4: 18 h, 5: 42 h after heat shock, and 6: adapted culture. FDR: false discovery rate, NA: no data available.
(XLSX)

S3 Table Oligonucleotid primers used for microarray validation by quantitative reverse transcriptase PCR. (DOCX)
