# Supplementary figures and images for: Heat shock and prolonged heat stress attenuate neurotoxin and sporulation gene expression in group I Clostridium botulinum strain ATCC 3502
Source: PLoS One. 2017 May 2;12(5):e0176944. doi: 10.1371/journal.pone.0176944 (PMC5413062; doi:10.1371/journal.pone.0176944)

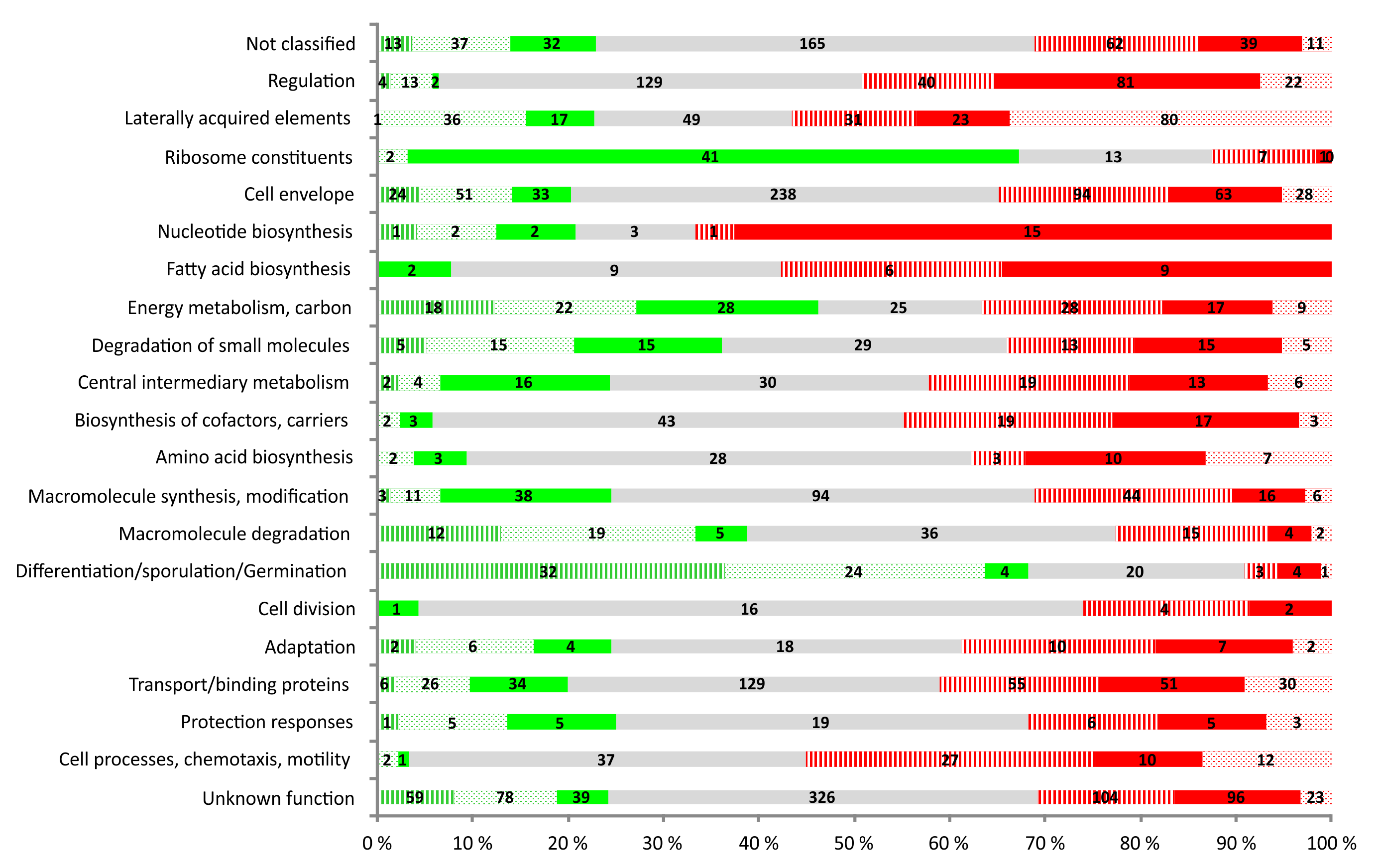

Supplement: S1 Fig — The clusters were created using the k-means clustering method with Euclidean distance and are shown in Fig 3. Numbers indicate the number of genes assigned to each cluster. Green striped: cluster 1, green dotted: cluster 2, green solid: cluster 3, grey: no cluster, red striped: cluster 4, red solid: cluster 5, red dotted: cluster 6. (TIF) [file pone.0176944.s001.tif]
