# Supplementary material for: Heat shock and prolonged heat stress attenuate neurotoxin and sporulation gene expression in group I Clostridium botulinum strain ATCC 3502
Source: PLoS One. 2017 May 2;12(5):e0176944. doi: 10.1371/journal.pone.0176944 (PMC5413062; doi:10.1371/journal.pone.0176944)
Supplement: S1 Table — Time points 1: immediately after heat shock, 2: 10 min, 3: 1 h, 4: 18 h, 5: 42 h after heat shock, and 6: adapted culture; values in bold: statistically significant change in expression (fold-change ≥ 2 or ≤ -2 and false discovery rate values [FDR] ≤ 0.05), values in italics: FDR values > 0.05; NA: no data available;–: no assignment to a cluster. (DOCX) [file pone.0176944.s002.docx]

|  |  |  |  |  | | | | | | |  | | | | | | |  | | | | | |  | | | | | |  | | | | | | |  | | |  |  |
| --- | --- | --- | --- | --- | --- | --- | --- | --- | --- | --- | --- | --- | --- | --- | --- | --- | --- | --- | --- | --- | --- | --- | --- | --- | --- | --- | --- | --- | --- | --- | --- | --- | --- | --- | --- | --- | --- | --- | --- | --- | --- |
|  |  |  |  |  | | | | | | |  | | | | | | |  | | | | | | Time point | | | | | | | | | | | | |  | | |  |  |
| Group | Gene |  | Cluster | Product | | | | | | | 1 | | | | 2 | | | | | | 3 | | | | | | 4 | | | | | 5 | | | | | 6 | | |  |  |
| Transcription and translation related genes | | | | | | | | |  | | | |  | | | |  | | | | | |  | | | | | |  | | | | | |  | | |  |  |  |  |
|  | *rpoA, cbo3452* | | 3 | DNA-directed RNA polymerase alpha chain | | | | | | | 1.4 | | | | -1.7 | | | | | | **-2.4** | | | | | | *-1.2* | | | | | -1.2 | | | | | *-1.1* | | |  |  |
|  | *rpoB, cbo3488* | | 3 | DNA-directed RNA polymerase beta chain | | | | | | | 1.3 | | | | **-4.1** | | | | | | **-2.6** | | | | | | -1.4 | | | | | -1.3 | | | | | *-1.2* | | |  |  |
|  | *rpoC, cbo3487* | | 3 | DNA-directed RNA polymerase beta' chain | | | | | | | 1.6 | | | | -1.9 | | | | | | **-2.4** | | | | | | -1.5 | | | | | -1.4 | | | | | *-1.3* | | |  |  |
|  | *deaD, cbo2802* | | 3 | ATP-dependent RNA helicase (cold-shock dead-box protein) | | | | | | | **-3.0** | | | | **-2.9** | | | | | | **-2.2** | | | | | | -1.8 | | | | | **-2.0** | | | | | *-1.5* | | |  |  |
|  | *infA, cbo3457* | | 3 | translation initiation factor IF-1 | | | | | | | 1.3 | | | | **-3.3** | | | | | | **-2.2** | | | | | | *-1.1* | | | | | *-1.1* | | | | | *-1.0* | | |  |  |
|  | *infC, cbo3137* | | 3 | translation initiation factor IF-3 | | | | | | | **-2.0** | | | | **-3.7** | | | | | | *-1.1* | | | | | | *1.4* | | | | | *1.1* | | | | | *1.1* | | |  |  |
|  | *efp, cbo1897* | | 4 | elongation factor P | | | | | | | **-2.9** | | | | **-2.8** | | | | | | 1.5 | | | | | | 1.9 | | | | | **2.1** | | | | | **2.2** | | |  |  |
|  | *cbo2434* | | 4 | elongation factor Ts | | | | | | | -1.5 | | | | **-3.2** | | | | | | 1.4 | | | | | | *1.3* | | | | | 1.4 | | | | | 1.5 | | |  |  |
|  | *tufA, cbo3482* | | 4 | elongation factor Tu | | | | | | | *1.1* | | | | **-2.1** | | | | | | *1.2* | | | | | | 1.6 | | | | | *1.2* | | | | | *1.3* | | |  |  |
|  | *fusA, cbo3483* | | 3 | translation elongation factor G, Ef-G | | | | | | | *-1.1* | | | | **-3.5** | | | | | | -1.9 | | | | | | *-1.1* | | | | | *-1.1* | | | | | *-1.1* | | |  |  |
|  | *tufB, cbo3496* | | 4 | elongation factor Tu | | | | | | | *1.1* | | | | **-2.0** | | | | | | *1.2* | | | | | | 1.5 | | | | | *1.2* | | | | | *1.2* | | |  |  |
|  | *sigA, cbo2938* | | 3 | RNA polymerase sigma factor RpoD | | | | | | | -1.7 | | | | -1.9 | | | | | | **-4.0** | | | | | | *1.4* | | | | | 1.8 | | | | | 1.8 | | |  |  |
| Class I heat shock genes | | | | |  | |  | | | |  | | | |  | | | | | |  | | | | | |  | | | |  |  |  |  |  |  |  |  |  |  |  |
|  | *dnaJ, cbo2958* | | 5 | Molecular chaperone DnaJ | | | | | | | 1.7 | | | | **2.4** | | | | | | **3.0** | | | | | | 1.4 | | | | | *-1.2* | | | | | *1.2* | | |  |  |
|  | *dnaK, cbo2959* | | 5 | Molecular chaperone DnaK | | | | | | | **2.8** | | | | **4.7** | | | | | | **5.1** | | | | | | 1.2 | | | | | *1.1* | | | | | 1.3 | | |  |  |
|  | *grpE, cbo2960* | | 5 | Heat shock protein GrpE | | | | | | | **3.8** | | | | **7.5** | | | | | | **7.6** | | | | | | 1.5 | | | | | 1.2 | | | | | 1.6 | | |  |  |
|  | *hrcA, cbo2961* | | 5 | Heat-inducible transcription repressor | | | | | | | **5.6** | | | | **8.7** | | | | | | **8.0** | | | | | | *1.2* | | | | | *1.0* | | | | | 1.4 | | |  |  |
|  | *groEL, cbo3298* | | 5 | Molecular chaperone GroEL | | | | | | | **3.3** | | | | **5.1** | | | | | | **8.4** | | | | | | **2.4** | | | | | 1.8 | | | | | **2.5** | | |  |  |
|  | *groES, cbo3299* | | 5 | Co-chaperonin GroES | | | | | | | **4.9** | | | | **7.1** | | | | | | **12.2** | | | | | | **2.2** | | | | | 1.4 | | | | | **2.1** | | |  |  |
| Class III heat shock genes | | | | | |  | | | |  | | | |  | | | | | |  | | | | | |  | | | | |  | | | | |  |  |  |  |  |  |
|  | *cbo3507* | | 5 | DNA integrity scanning protein DisA | | | | | | | 1.3 | | | | **5.2** | | | | | | **6.2** | | | | | | 1.9 | | | | | 1.6 | | | | | 1.6 | | |  |  |
|  | *radA, cbo3508* | | 6 | DNA repair protein RadA, | | | | | | | **2.9** | | | | **9.0** | | | | | | **11.3** | | | | | | **3.1** | | | | | **2.4** | | | | | **2.6** | | |  |  |
|  | *clpC, cbo3509* | | 6 | negative regulator of genetic competence MecB/ClpC | | | | | | | **4.1** | | | | **17.0** | | | | | | **18.4** | | | | | | **3.9** | | | | | **2.8** | | | | | **2.4** | | |  |  |
|  | *cbo3510* | | 6 | ATP:guanido phosphotransferase | | | | | | | **11.4** | | | | **38.3** | | | | | | **37.5** | | | | | | **4.5** | | | | | **3.5** | | | | | **4.1** | | |  |  |
|  | *cbo3511* | | 6 | UVR domain protein | | | | | | | **12.2** | | | | **33.5** | | | | | | **34.5** | | | | | | **4.5** | | | | | **2.9** | | | | | **3.5** | | |  |  |
|  | *ctsR, cbo3512* | | 6 | Transciptional repressor CtsR | | | | | | | **13.9** | | | | **45.7** | | | | | | **42.8** | | | | | | **4.5** | | | | | **3.0** | | | | | **4.0** | | |  |  |
|  |  | |  |  | | | | | | |  | | | |  | | | | | |  | | | | | |  | | | | |  | | | | |  | | |  |  |
|  | *clpP, cbo3231* | | - | ATP-dependent Clp protease proteolytic subunit | | | | | | | 1.4 | | | | 1.2 | | | | | | 1.1 | | | | | | *-1.0* | | | | | *-1.1* | | | | | *-1.1* | | |  |  |
| Other heat stress related genes | | | | | | | |  | | | |  | | | |  | | | | | |  | | | | | |  | | | | | |  | | |  |  |  |  |  |
|  | *htpG, cbo1985* | | 5 | Heat shock protein 90 | | | | | | | **4.5** | | | | **8.5** | | | | | | **12.0** | | | | | | 1.6 | | | | | **2.2** | | | | | **2.5** | | |  |  |
|  | *cbo1760* | | 5 | Heat shock protein | | | | | | | **2.2** | | | | **5.7** | | | | | | **9.5** | | | | | | **2.0** | | | | | *-1.1* | | | | | 1.6 | | |  |  |
|  | *clpB, cbo0409* | | 5 | ClpA-type chaperone | | | | | | | 1.5 | | | | **2.2** | | | | | | 1.8 | | | | | | 1.8 | | | | | **2.3** | | | | | **2.9** | | |  |  |
|  | *cbo0831* | | 2 | Heat shock protein | | | | | | | *1.1* | | | | 1.2 | | | | | | -1.2 | | | | | | **-2.5** | | | | | **-2.0** | | | | | **-2.9** | | |  |  |
|  | *clpX, cbo3230* | | - | ATP-dependent protease ATP-binding subunit ClpX | | | | | | | 1.3 | | | | *1.1* | | | | | | -1.0 | | | | | | -1.3 | | | | | -1.4 | | | | | -1.3 | | |  |  |
|  | *lonB, cbo3229* | | 2 | ATP-dependent protease | | | | | | | *1.1* | | | | *1.2* | | | | | | **-4.2** | | | | | | **-4.3** | | | | | **-5.1** | | | | | -1.5 | | |  |  |
|  | *cbo2806* | | 5 | Universal stress protein family protein | | | | | | | 1.5 | | | | **2.0** | | | | | | **2.3** | | | | | | -1.3 | | | | | *-1.1* | | | | | -1.4 | | |  |  |
| SOS reponse | |  |  |  | | | | | | |  | | | |  | | | | | |  | | | | | |  | | | | |  | | | | |  | | |  |  |
|  | *recA, cbo2405* | | 5 | RecA protein | | | | | | | *1.0* | | | | 1.9 | | | | | | **2.9** | | | | | | 1.8 | | | | | **2.2** | | | | | 1.8 | | |  |  |
|  | *dinB, cbo2818* | | 5 | DNA polymerase IV | | | | | | | 1.2 | | | | 1.8 | | | | | | **2.4** | | | | | | *1.5* | | | | | 1.5 | | | | | *1.8* | | |  |  |
|  | *pcrA, cbo3271* | | 4 | ATP-dependent DNA helicase | | | | | | | *-1.1* | | | | 1.4 | | | | | | 1.7 | | | | | | 1.7 | | | | | **2.0** | | | | | **2.8** | | |  |  |
|  | *ligA, cbo3270* | | 4 | DNA ligase | | | | | | | *1.1* | | | | *1.1* | | | | | | 1.5 | | | | | | **2.5** | | | | | 1.9 | | | | | **2.6** | | |  |  |
|  | *uvrC, cbo3380* | | 4 | Excinuclease ABC subunit C | | | | | | | *1.1* | | | | 1.3 | | | | | | *1.0* | | | | | | **2.5** | | | | | **4.3** | | | | | **4.1** | | |  |  |
|  | *dnaE, cbo3374* | | 4 | DNA polymerase III alpha subunit | | | | | | | *-1.0* | | | | -1.5 | | | | | | 1.2 | | | | | | 1.6 | | | | | 1.4 | | | | | **2.0** | | |  |  |
|  | *dnaE, cboP01* | | 4 | DNA polymerase III alpha subunit | | | | | | | -1.8 | | | | -1.4 | | | | | | *1.2* | | | | | | **2.2** | | | | | **2.8** | | | | | **3.2** | | |  |  |
| Cold stress response | |  |  |  | | | | | | |  | | | |  | | | | | |  | | | | | |  | | | | |  | | | | |  | | |  |  |
|  | *cspC, cbo1772* | | 2 | Cold shock protein C | | | | | | | -1.6 | | | | **-2.1** | | | | | | **-3.1** | | | | | | **-5.7** | | | | | **-6.2** | | | | | ***-3.6*** | | |  |  |
|  | *cspA, cbo0282* | | - | Cold shock protein A | | | | | | | *-1.3* | | | | *1.2* | | | | | | *1.5* | | | | | | *1.2* | | | | | *1.1* | | | | | *1.3* | | |  |  |
|  | *cspB, cbo1387* | | - | Cold shock protein B | | | | | | | *-1.1* | | | | *1.0* | | | | | | *1.0* | | | | | | -1.6 | | | | | -1.3 | | | | | -1.7 | | |  |  |
| Neurotoxin cluster (NTC) | | | | | | | | | | |  | | | |  | | | | | |  | | | | | |  | | | | |  | | | | |  | | |  |  |
|  | *ha70, cbo0801* | | 2 | Hemagglutinin component | | | | | | | *1.0* | | | | -1.4 | | | | | | **-7.1** | | | | | | **-4.6** | | | | | **-4.0** | | | | | **-6.0** | | |  |  |
|  | *ha17, cbo0802* | | 2 | Hemagglutinin component | | | | | | | *-1.0* | | | | **-2.0** | | | | | | **-7.5** | | | | | | **-4.1** | | | | | **-4.0** | | | | | **-6.8** | | |  |  |
|  | *ha33, cbo0803* | | 2 | Hemagglutinin component | | | | | | | *-1.1* | | | | **-2.0** | | | | | | **-6.7** | | | | | | **-4.0** | | | | | **-4.0** | | | | | **-5.4** | | |  |  |
|  | *ntnH, cbo0805* | | 2 | Nontoxic-nonhemagglutinin component | | | | | | | *1.1* | | | | **-2.7** | | | | | | **-5.2** | | | | | | **-3.7** | | | | | **-3.6** | | | | | **-5.5** | | |  |  |
|  | *botA, cbo0806* | | 2 | Botulinum neurotoxin type A | | | | | | | *1.0* | | | | **-2.2** | | | | | | **-5.4** | | | | | | **-3.0** | | | | | **-3.6** | | | | | **-5.1** | | |  |  |
|  | *botR, cbo0804* | | 5 | Transcriptional regulator BotR | | | | | | | 1.6 | | | | NA | | | | | | 1.8 | | | | | | NA | | | | | **2.2** | | | | | NA | | |  |  |
| Toxin related | | |  |  | | | | | | |  | | | |  | | | | | |  | | | | | |  | | | | |  | | | | |  | | |  |  |
|  | *codY, cbo2436* | | - | Ttranscriptional repressor CodY | | | | | | | -1.3 | | | | *1.1* | | | | | | 1.9 | | | | | | *-1.1* | | | | | *1.1* | | | | | *-1.0* | | |  |  |
|  | *cbo0353* | | - | Sensor histidine kinase | | | | | | | -1.8 | | | | -1.6 | | | | | | *-1.2* | | | | | | *-1.5* | | | | | -1.5 | | | | | *-1.4* | | |  |  |
|  | *cbo0352* | | 2 | DNA-binding response regulator | | | | | | | -1.8 | | | | -1.7 | | | | | | -1.5 | | | | | | **-2.3** | | | | | **-2.6** | | | | | **-3.4** | | |  |  |
|  |  | |  |  | | | | | | |  | | | |  | | | | | |  | | | | | |  | | | | |  | | | | |  | | |  |  |
| Sporulation regulation related genes | | | | | | | |  | | | |  | | | |  | | | | | |  | | | | | |  | | | | | |  | | |  |  |  |  |  |
|  | *sigH, cbo3497* | | - | RNA polymerase factor sigma-70 | | | | | | | -1.5 | | | | -1.4 | | | | | | 1.9 | | | | | | *1.4* | | | | | 1.5 | | | | | 1.4 | | |  |  |
|  | *sigF, cbo3087* | | 2 | Sporulation sigma factor SigF | | | | | | | *1.1* | | | | *1.0* | | | | | | -1.2 | | | | | | *-1.2* | | | | | -1.8 | | | | | **-2.0** | | |  |  |
|  | *sigG, cbo2532* | | 1 | Sporulation sigma factor SigG | | | | | | | 1.2 | | | | 1.4 | | | | | | *1.1* | | | | | | **-11.3** | | | | | **-17.5** | | | | | **-28.1** | | |  |  |
|  | *sigE, cbo2533* | | 2 | Sporulation sigma factor SigE | | | | | | | 1.3 | | | | 1.7 | | | | | | 1.8 | | | | | | **-7.2** | | | | | **-7.8** | | | | | **-4.8** | | |  |  |
|  | *sigK, cbo2541* | | 1 | Sporulation sigma factor SigK | | | | | | | *-1.1* | | | | *1.1* | | | | | | **-2.6** | | | | | | **-14.4** | | | | | **-27.1** | | | | | **-40.3** | | |  |  |
|  | *spo0A, cbo1872* | | 3 | Sporulation transcription factor Spo0A | | | | | | | **-2.0** | | | | -1.2 | | | | | | 1.2 | | | | | | 1.4 | | | | | -1.5 | | | | | **-2.5** | | |  |  |
|  | *pemK, cbo3395A* | | 2 | PemK family protein | | | | | | | *1.1* | | | | 1.3 | | | | | | 1.3 | | | | | | **-2.3** | | | | | **-6.7** | | | | | **-23.6** | | |  |  |
|  | *cbo3396* | | 1 | Putative CopG-family transcriptional regulator | | | | | | | *1.1* | | | | 1.4 | | | | | | 1.3 | | | | | | **-2.6** | | | | | **-8.5** | | | | | **-23.3** | | |  |  |
|  | *cbo0900* | | 1 | Unknown function | | | | | | | *1.1* | | | | -1.2 | | | | | | 1.5 | | | | | | **-8.7** | | | | | **-34.4** | | | | | **-118.4** | | |  |  |
|  |  | |  |  | | | | | | |  | | | |  | | | | | |  | | | | | |  | | | | |  | | | | |  | | |  |  |
|  | *cbo1594* | | 1 | Unknown function | | | | | | | *1.0* | | | | 1.3 | | | | | | 1.4 | | | | | | **-4.9** | | | | | **-14.0** | | | | | **-70.7** | | |  |  |
|  | *cbo2267* | | 1 | Unknown function | | | | | | | *-1.0* | | | | *1.3* | | | | | | *1.4* | | | | | | **-4.0** | | | | | **-10.8** | | | | | **-72.7** | | |  |  |
|  | *cbo2273* | | 1 | Unknown function | | | | | | | *1.0* | | | | *1.4* | | | | | | *1.4* | | | | | | **-4.0** | | | | | **-8.5** | | | | | **-72.6** | | |  |  |
|  | *disA, cbo3507* | | 5 | DNA integrity scanning protein DisA | | | | | | | 1.3 | | | | **5.2** | | | | | | **6.2** | | | | | | 1.9 | | | | | 1.6 | | | | | 1.6 | | |  |  |
| Chemotaxis and mobility related genes | | | | | | | |  | | | |  | | | |  | | | | | |  | | | | | |  | | | | | |  | | |  |  |  |  |  |
|  | *sigD, cbo2641* | | 4 | RNA polymerase sigma factor | | | | | | | -1.3 | | | | -1.5 | | | | | | **-2.1** | | | | | | 1.7 | | | | | **2.6** | | | | | 1.7 | | |  |  |
|  | *cbo0242* | | 5 | Flagellin | | | | | | | 1.0 | | | | 1.8 | | | | | | **2.0** | | | | | | *1.0* | | | | | -1.4 | | | | | -1.6 | | |  |  |
|  | *cbo2666* | | 4 | Flagellin | | | | | | | -1.8 | | | | -1.6 | | | | | | *1.1* | | | | | | **2.0** | | | | | **2.0** | | | | | 1.8 | | |  |  |
|  | *cbo2695* | | - | Flagellin | | | | | | | *-1.0* | | | | -1.4 | | | | | | *-1.1* | | | | | | 1.7 | | | | | 1.6 | | | | | *1.1* | | |  |  |
|  | *cbo2730* | | 4 | Flagellin | | | | | | | *-1.0* | | | | -1.6 | | | | | | 1.3 | | | | | | **3.0** | | | | | **2.2** | | | | | 1.4 | | |  |  |
|  | *cbo2731* | | 4 | Flagellin | | | | | | | *1.0* | | | | -1.6 | | | | | | 1.3 | | | | | | **3.2** | | | | | **2.1** | | | | | 1.4 | | |  |  |
| Phage related | |  |  |  | | | | | | |  | | | |  | | | | | |  | | | | | |  | | | | |  | | | | |  | | |  |  |
|  | *cbo2376* | | 6 | RNA polymerase sigma factor | | | | | | | 1.4 | | | | **2.1** | | | | | | **3.4** | | | | | | **5.5** | | | | | **2.9** | | | | | **2.0** | | |  |  |
|  | *cbo2374* | | 6 | SpoVT/AbrB family regulatory protein | | | | | | | 1.3 | | | | 1.8 | | | | | | **3.2** | | | | | | **5.8** | | | | | **2.7** | | | | | *1.4* | | |  |  |
|  | *cbo2389* | | 6 | Phage associated-antirepressor | | | | | | | 1.8 | | | | **3.1** | | | | | | **3.6** | | | | | | **5.9** | | | | | **4.2** | | | | | **2.1** | | |  |  |
|  | *cbo2390* | | 6 | Transcriptional regulator | | | | | | | 1.8 | | | | **3.1** | | | | | | **3.3** | | | | | | **5.5** | | | | | **3.8** | | | | | **2.1** | | |  |  |
| Carbon metabolism | |  |  |  | | | | | | |  | | | |  | | | | | |  | | | | | |  | | | | |  | | | | |  | | |  |  |
|  | *cbo3197* | | 1 | Electron transfer flavoprotein subunit alpha | | | | | | | *-1.1* | | | | -1.6 | | | | | | **-17.2** | | | | | | **-35.3** | | | | | **-49.9** | | | | | **-19.5** | | |  |  |
|  | *cbo3198* | | 1 | Electron transfer flavoprotein subunit alpha | | | | | | | *-1.1* | | | | -1.9 | | | | | | **-18.3** | | | | | | **-32.9** | | | | | **-48.1** | | | | | **-18.9** | | |  |  |
|  | *bcd, cbo3199* | | 1 | Butyryl-CoA dehydrogenase | | | | | | | *-1.1* | | | | **-2.1** | | | | | | **-19.2** | | | | | | **-34.4** | | | | | **-52.8** | | | | | **-24.3** | | |  |  |
|  | *thl, cbo3200* | | 1 | Acetyl-CoA acetyltransferase | | | | | | | *-1.0* | | | | -1.5 | | | | | | **-15.0** | | | | | | **-33.5** | | | | | **-52.4** | | | | | **-20.5** | | |  |  |
|  | *hbd, cbo3201* | | 1 | 3-hydroxybutyryl-CoA dehydrogenase | | | | | | | *-1.1* | | | | -1.8 | | | | | | **-17.0** | | | | | | **-35.6** | | | | | **-53.0** | | | | | **-17.1** | | |  |  |
|  | *crt, cbo3202* | | 1 | 3-hydroxybutyryl-CoA dehydratase | | | | | | | **-2.1** | | | | **-6.4** | | | | | | **-24.1** | | | | | | **-36.2** | | | | | **-62.7** | | | | | **-24.2** | | |  |  |
|  | *adhE, cbo0345* | | 6 | Aldehyde-alcohol dehydrogenase | | | | | | | 1.7 | | | | 1.8 | | | | | | **5.0** | | | | | | **4.0** | | | | | **2.7** | | | | | **3.0** | | |  |  |
|  | *glpK, cbo2784* | | 5 | Glycerol kinase | | | | | | | **2.1** | | | | **5.9** | | | | | | **-2.2** | | | | | | **-2.8** | | | | | *1.1* | | | | | *1.4* | | |  |  |
|  | *glpA, cbo1068* | | 5 | FAD-dependent oxidoreductase | | | | | | | **6.1** | | | | **8.3** | | | | | | 1.6 | | | | | | *-1.4* | | | | | *1.6* | | | | | **2.5** | | |  |  |
|  | *glpF, cbo2785* | | 5 | Glycerol uptake facilitator protein | | | | | | | **2.8** | | | | **6.3** | | | | | | -1.6 | | | | | | **-3.2** | | | | | *1.4* | | | | | 1.5 | | |  |  |
|  | *glpT, cbo1071* | | 6 | Glycerol-3-phosphate transporter | | | | | | | **2.7** | | | | **16.4** | | | | | | 1.6 | | | | | | **2.6** | | | | | **4.6** | | | | | **7.1** | | |  |  |
|  | *treB, cbo1987* | | 5 | PTS system, trehalose-specific IIbc component | | | | | | | 1.6 | | | | **4.7** | | | | | | **4.1** | | | | | | **2.1** | | | | | **3.0** | | | | | **2.0** | | |  |  |
|  | *treA, cbo1988* | | 5 | Alpha,alpha-phosphotrehalase | | | | | | | **2.1** | | | | **4.7** | | | | | | **3.7** | | | | | | 1.6 | | | | | 1.8 | | | | | 1.8 | | |  |  |
|  | *treR, cbo1989* | | 5 | Trehalose operon repressor | | | | | | | 1.7 | | | | **2.8** | | | | | | **2.8** | | | | | | *1.2* | | | | | 1.4 | | | | | 1.5 | | |  |  |
|  | *srlD, cbo3413* | | 5 | Sorbitol-6-phosphate dehydrogenase | | | | | | | *-1.0* | | | | **4.8** | | | | | | **3.2** | | | | | | **2.1** | | | | | **2.7** | | | | | **2.5** | | |  |  |
|  | *srlB, cbo3414* | | 6 | PTS system glucitol/sorbitol-specific transporter Subunit IIA | | | | | | | 1.8 | | | | **10.7** | | | | | | **2.2** | | | | | | **3.7** | | | | | **3.7** | | | | | **3.5** | | |  |  |
|  | *srlE, cbo3415* | | 5 | PTS system glucitol/sorbitol-specific transporter Subunit IIBC | | | | | | | 1.6 | | | | **7.0** | | | | | | 1.6 | | | | | | **2.8** | | | | | **2.3** | | | | | **2.2** | | |  |  |
|  | *srlA, cbo3416* | | 6 | PTS system glucitol/sorbitol-specific transporter Subunit IIC | | | | | | | 1.9 | | | | **8.6** | | | | | | **2.1** | | | | | | **3.5** | | | | | **2.6** | | | | | **2.6** | | |  |  |
|  | *srlM, cbo3417* | | 5 | Glucitol operon activator protein | | | | | | | 1.8 | | | | **4.7** | | | | | | **2.1** | | | | | | **2.9** | | | | | **2.4** | | | | | **2.1** | | |  |  |
|  | *cbo0417* | | 6 | HMGL-related enzyme | | | | | | | 1.7 | | | | **3.0** | | | | | | **2.0** | | | | | | **3.8** | | | | | **5.1** | | | | | **20.2** | | |  |  |
|  | *cbo0418* | | 6 | Aconitate hydratase | | | | | | | 1.3 | | | | **2.4** | | | | | | **2.1** | | | | | | **4.1** | | | | | **5.9** | | | | | **22.0** | | |  |  |
|  | *cbo0419* | | 6 | Isocitrate/isopropylmalate family dehydrogenase | | | | | | | 1.2 | | | | **2.3** | | | | | | 1.6 | | | | | | **3.7** | | | | | **5.5** | | | | | **20.7** | | |  |  |
| Protein and amino acid metabolism | | | | | | | | |  | | | |  | | | |  | | | | | |  | | | | | |  | | | | | |  | | |  |  |  |  |
|  | *cbo1442* | | 1 | Secreted protease | | | | | | | *1.1* | | | | -1.7 | | | | | | **-3.2** | | | | | | **-4.3** | | | | | **-8.4** | | | | | **-11.6** | | |  |  |
|  | *cbo1443* | | 2 | Secreted protease | | | | | | | *-1.2* | | | | -1.7 | | | | | | **-3.8** | | | | | | **-2.5** | | | | | **-5.9** | | | | | **-13.6** | | |  |  |
|  | *cbo1444* | | 2 | Secreted protease | | | | | | | *-1.1* | | | | -1.5 | | | | | | **-3.2** | | | | | | **-2.4** | | | | | **-5.3** | | | | | **-13.4** | | |  |  |
|  | *cbo1445* | | 2 | Secreted protease | | | | | | | *-1.1* | | | | -1.3 | | | | | | **-3.0** | | | | | | **-2.8** | | | | | **-5.9** | | | | | **-13.9** | | |  |  |
|  | *cbo1446* | | 2 | Secreted protease | | | | | | | *-1.0* | | | | -1.3 | | | | | | **-2.8** | | | | | | **-3.1** | | | | | **-6.1** | | | | | **-14.9** | | |  |  |
|  | *cbo1439* | | 1 | ATP-dependent protease | | | | | | | *1.1* | | | | -1.1 | | | | | | -1.6 | | | | | | **-4.2** | | | | | **-10.7** | | | | | **-14.6** | | |  |  |
|  | *cbo0161* | | 1 | Peptidase | | | | | | | *1.1* | | | | *1.1* | | | | | | **-7.4** | | | | | | **-33.2** | | | | | **-31.6** | | | | | **-68.2** | | |  |  |
|  | *grdX, cbo1255* | | 6 | GrdX protein | | | | | | | -1.3 | | | | 1.9 | | | | | | **5.9** | | | | | | **3.8** | | | | | **4.6** | | | | | **3.8** | | |  |  |
|  | *grdE, cbo1256* | | 4 | Glycine reductase complex component B, alpha and beta | | | | | | | **-2.0** | | | | **-2.4** | | | | | | -1.5 | | | | | | 1.9 | | | | | **2.4** | | | | | 1.6 | | |  |  |
|  | *grdB, cbo1257* | | 3 | Glycine reductase complex component B gamma subunit | | | | | | | -1.9 | | | | **-3.4** | | | | | | **-4.3** | | | | | | 1.5 | | | | | 1.7 | | | | | 1.3 | | |  |  |
|  | *trxB2, cbo1259* | | 3 | Thioredoxin-disulfide reductase | | | | | | | -1.8 | | | | **-3.6** | | | | | | **-5.3** | | | | | | *1.2* | | | | | 1.4 | | | | | 1.4 | | |  |  |
|  | *trxA, cbo1260* | | 3 | Thioredoxin family protein | | | | | | | -1.8 | | | | **-3.4** | | | | | | **-6.5** | | | | | | *1.1* | | | | | 1.4 | | | | | 1.2 | | |  |  |
|  | *grdA, cbo1261* | | 3 | Glycine/sarcosine/betaine reductase complex protein A | | | | | | | -1.8 | | | | **-3.2** | | | | | | **-5.6** | | | | | | *1.3* | | | | | 1.3 | | | | | *1.3* | | |  |  |
|  | *grdC, cbo1263* | | 3 | Glycine reductase complex component C subunit beta | | | | | | | -1.5 | | | | **-3.2** | | | | | | **-6.1** | | | | | | 1.3 | | | | | 1.3 | | | | | 1.3 | | |  |  |
|  | *grdD, cbo1264* | | 3 | Glycine reductase complex component C subunit alpha | | | | | | | -1.4 | | | | **-3.1** | | | | | | **-7.1** | | | | | | *1.2* | | | | | 1.4 | | | | | 1.3 | | |  |  |
|  | *prdB2, cbo2461* | | 4 | Proline reductase | | | | | | | *-1.0* | | | | -1.3 | | | | | | -1.6 | | | | | | 1.4 | | | | | **5.9** | | | | | **7.4** | | |  |  |
|  | *prdA1, cbo2464* | | 4 | Proline reductase | | | | | | | *-1.1* | | | | 1.5 | | | | | | 1.4 | | | | | | **2.2** | | | | | **3.5** | | | | | **3.5** | | |  |  |
|  | *prdF, cbo2474* | | 6 | Proline racemase | | | | | | | 1.2 | | | | **2.8** | | | | | | 1.7 | | | | | | **2.9** | | | | | **4.1** | | | | | **3.7** | | |  |  |
|  | *prdE, cbo2475* | | 6 | PrdE protein | | | | | | | 1.9 | | | | **4.3** | | | | | | **2.5** | | | | | | **3.5** | | | | | **4.6** | | | | | **4.7** | | |  |  |
|  | *prdD, cbo2476* | | 6 | PrdD protein | | | | | | | 1.9 | | | | **4.1** | | | | | | **2.5** | | | | | | **3.0** | | | | | **4.3** | | | | | **4.5** | | |  |  |
|  | *prdB2, cbo2477* | | 4 | Subunit of proline reductase | | | | | | | *-1.0* | | | | 1.8 | | | | | | 1.8 | | | | | | **2.7** | | | | | 1.7 | | | | | 1.9 | | |  |  |
|  | *prdA2, cbo2480* | | 5 | Subunit of proline reductase | | | | | | | *-1.1* | | | | **2.1** | | | | | | 1.9 | | | | | | **2.7** | | | | | 1.4 | | | | | *1.3* | | |  |  |
|  | *prdC2, cbo2482* | | 5 | Electron transferring subunit of proline reductase | | | | | | | *1.2* | | | | **2.4** | | | | | | 1.6 | | | | | | **2.4** | | | | | 1.5 | | | | | 1.4 | | |  |  |
|  | *hadI, cbo2192* | | 1 | Activator of 2-hydroxyisocaproyl-CoA dehydratase | | | | | | | *1.0* | | | | *1.1* | | | | | | **-5.1** | | | | | | **-13.9** | | | | | **-15.3** | | | | | **-9.5** | | |  |  |
|  | *cbo2193* | | 2 | Putative membrane protein | | | | | | | -1.2 | | | | *-1.1* | | | | | | **-5.8** | | | | | | **-8.4** | | | | | **-7.8** | | | | | **-5.8** | | |  |  |
|  | *etfA1, cbo2194* | | 1 | Electron transfer flavoprotein subunit alpha | | | | | | | *-1.1* | | | | 1.3 | | | | | | **-5.7** | | | | | | **-19.5** | | | | | **-18.6** | | | | | **-26.2** | | |  |  |
|  | *etfB1, cbo2195* | | 1 | Electron transfer flavoprotein subunit beta | | | | | | | *1.0* | | | | 1.4 | | | | | | **-5.1** | | | | | | **-23.4** | | | | | **-16.2** | | | | | **-25.2** | | |  |  |
|  | *acdB, cbo2196* | | 1 | Acyl-CoA dehydrogenase, short-chain specific | | | | | | | *1.1* | | | | 1.4 | | | | | | **-5.0** | | | | | | **-20.8** | | | | | **-15.0** | | | | | **-24.9** | | |  |  |
|  | *hadC, cbo2197* | | 1 | Subunit of oxygen-sensitive 2-hydroxyisocaproyl-CoA dehydratase | | | | | | | *1.1* | | | | 1.4 | | | | | | **-5.2** | | | | | | **-32.6** | | | | | **-21.0** | | | | | **-37.9** | | |  |  |
|  | *hadB, cbo2198* | | 1 | Subunit of oxygen-sensitive 2-hydroxyisocaproyl-CoA dehydratase | | | | | | | *1.1* | | | | 1.4 | | | | | | **-5.0** | | | | | | **-24.3** | | | | | **-18.1** | | | | | **-31.3** | | |  |  |
|  | *hadA, cbo2199* | | 1 | Isocaprenoyl-CoA:2-hydroxyisocaproate CoA-transferase | | | | | | | *-1.0* | | | | 1.3 | | | | | | **-4.5** | | | | | | **-26.7** | | | | | **-17.2** | | | | | **-33.0** | | |  |  |
|  | *fldC, cbo3289* | | 3 | R-phenyllactate dehydratase small subunit | | | | | | | **-2.0** | | | | **-2.9** | | | | | | **-9.6** | | | | | | *1.2* | | | | | **2.5** | | | | | 1.9 | | |  |  |
|  | *fldB, cbo3290* | | 3 | R-phenyllactate dehydratase medium subunit | | | | | | | -1.9 | | | | **-3.0** | | | | | | **-8.1** | | | | | | 1.3 | | | | | **2.4** | | | | | **2.0** | | |  |  |
|  | *fldI, cbo3291* | | 3 | R-phenyllactate dehydratase activator | | | | | | | -1.9 | | | | **-3.1** | | | | | | **-6.6** | | | | | | 1.4 | | | | | **2.6** | | | | | **2.2** | | |  |  |
|  | *fldA, cbo3292* | | 3 | E-cinnamoyl-CoA:R-phenyllactate CoA transferase | | | | | | | -1.9 | | | | **-2.9** | | | | | | **-6.4** | | | | | | 1.4 | | | | | **2.7** | | | | | **2.4** | | |  |  |
|  | *fldL, cbo3293* | | 3 | Putative CoA-ligase | | | | | | | -1.4 | | | | **-2.8** | | | | | | **-4.9** | | | | | | 1.3 | | | | | **3.4** | | | | | **2.8** | | |  |  |
|  | *luxS, cbo0725* | | 5 | S-ribosylhomocysteinase | | | | | | | **2.9** | | | | **7.6** | | | | | | **6.4** | | | | | | *1.1* | | | | | **-2.3** | | | | | **-3.5** | | |  |  |
|  | *metC, cbo0726* | | 5 | Cystathionine beta-lyase | | | | | | | **3.0** | | | | **8.0** | | | | | | **6.9** | | | | | | *1.1* | | | | | **-2.3** | | | | | **-3.4** | | |  |  |
|  | *metA, cbo1874* | | 5 | Homoserine O-succinyltransferase | | | | | | | 1.7 | | | | **2.6** | | | | | | **3.2** | | | | | | **2.4** | | | | | 1.9 | | | | | **2.2** | | |  |  |
|  | *hom, cbo1875* | | 6 | Homoserine dehydrogenase | | | | | | | 1.8 | | | | **3.4** | | | | | | **4.6** | | | | | | **3.7** | | | | | **3.8** | | | | | **3.4** | | |  |  |
|  | *metN2, cbo1499* | | 6 | D-methionine ABC transporter ATP-binding protein | | | | | | | **-2.3** | | | | NA | | | | | | 1.4 | | | | | | NA | | | | | **10.6** | | | | | **13.3** | | |  |  |
|  | *metI2, cbo1500* | | 6 | Methionine ABC transporter permease | | | | | | | -1.9 | | | | NA | | | | | | NA | | | | | | NA | | | | | **11.0** | | | | | **15.1** | | |  |  |
|  | *metQ3, cbo1501* | | 4 | D-methionine ABC transporter, substrate-binding lipoprotein | | | | | | | -1.8 | | | | *-2.3* | | | | | | *-1.4* | | | | | | 1.6 | | | | | **8.9** | | | | | **8.9** | | |  |  |
|  |  | |  |  | | | | | | |  | | | |  | | | | | |  | | | | | |  | | | | |  | | | | |  | | |  |  |
|  |  | |  |  | | | | | | |  | | | |  | | | | | |  | | | | | |  | | | | |  | | | | |  | | |  |  |
|  |  | |  |  | | | | | | |  | | | |  | | | | | |  | | | | | |  | | | | |  | | | | |  | | |  |  |
|  |  | |  |  | | | | | | |  | | | |  | | | | | |  | | | | | |  | | | | |  | | | | |  | | |  |  |
|  |  |  |  |  | | | | | | |  | | | | | | | |  | | | | | |  | | | | | | | |  | | | | | |  | |  |
|  |  |  |  |  | | | | | | |  | | | | | | | |  | | | | | |  | | | | | | | |  | | | | | |  | |  |
