# Supplementary material for: Heat shock and prolonged heat stress attenuate neurotoxin and sporulation gene expression in group I Clostridium botulinum strain ATCC 3502
Source: PLoS One. 2017 May 2;12(5):e0176944. doi: 10.1371/journal.pone.0176944 (PMC5413062; doi:10.1371/journal.pone.0176944)
Supplement: S3 Table — (DOCX) [file pone.0176944.s004.docx]

| Primer name | Sequence (5’ – 3’) |
| --- | --- |
| 16S rrn-F | AGCGGTGAAATGCGTAGAGA |
| 16S rrn -R | GGCACAGGGGGAGTTGATAC |
| botA-F | CGCGAAATGGTTATGGCTCT |
| botA-R | GCCTGCACCTAAAAGAGGATTT |
| ha33-F | CATCTCCTGTAAGGCCGATACTAA |
| ha33-R | GCATTTGAATCTTGTTGCGTTG |
| hrcA-F | ATAGTGGAGAACCCGTTGGA |
| hrcA-R | CCATATCTTCTAGGTCTGCCATCT |
| groES-F | CCTGGTGGATTAGTTGATGGA |
| groES-R | TCACTTCATTACCGGCATATTTT |
| sigE-F | AGCAACCTATGCTTCAAGATGT |
| sigE-R | AGCTCATTTCCATCCCAATC |
| sigK-F | ACTTATGGGATGTACTAGGAAGTG |
| sigK-R | TTCTTCTTCATCACTTAGAGGCTT |
| sigD-F | CCCATATCCAAAGGGGCTATGGA |
| sigD-R | AGTGGTACCTCATCTTCTGACGA |
| cheA-F | TGGTGGCGGTGTACCTGTTGA |
| cheA-R | AGGAGCCCTTTCCTTCTTCGCT |
| flgE-F | AGTGCCACAGGACCAAGTGCT |
| flgE-R | GCCTGTGCATCATCAGTGCCC |
| cbo3199-F | GGAATACGGTGGAGCTGGTA |
| cbo3199-R | TGGAGCGCAACATAAAGATG |
| cbo3202-F | AGCCGACTATAGCAGCCGTA |
| cbo3202-R | TCCTCCGAATCCTGGAGTTA |
|  |  |
|  |  |
|  |  |
|  |  |
|  |  |
|  |  |
|  |  |
